# Permafrost Thaw Increases Methylmercury Formation in Subarctic Fennoscandia

**DOI:** 10.1021/acs.est.0c04108

**Published:** 2021-04-27

**Authors:** Brittany Tarbier, Gustaf Hugelius, Anna Britta Kristina Sannel, Carluvy Baptista-Salazar, Sofi Jonsson

**Affiliations:** † Department of Physical Geography, Stockholm University, Stockholm 106 91, Sweden; ‡ Bolin Centre for Climate Research, Stockholm University, Stockholm 106 91, Sweden; § Department of Environmental Science, Stockholm University, Stockholm 106 91, Sweden

## Abstract

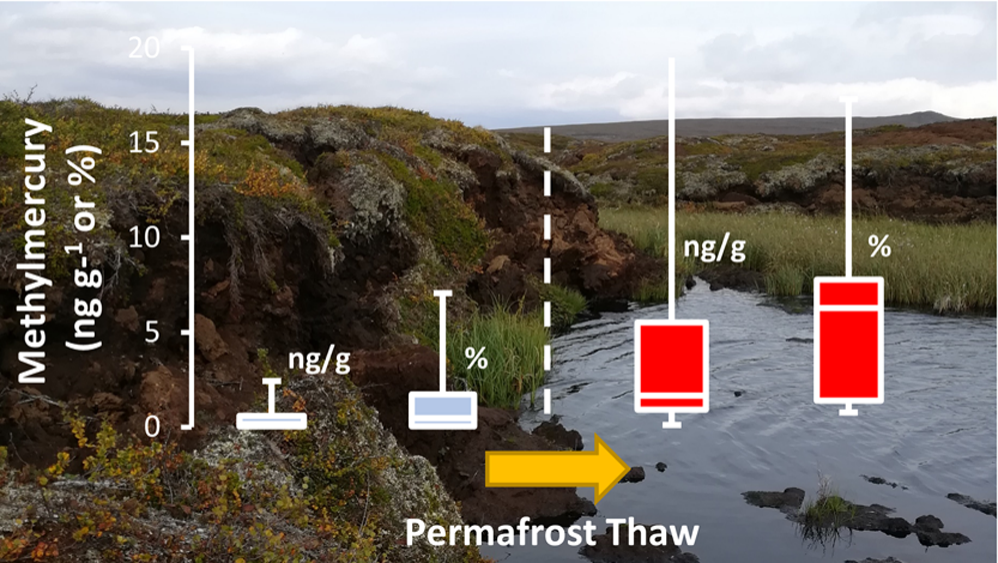

Methylmercury (MeHg) forms in anoxic environments and can bioaccumulate and biomagnify in aquatic food webs to concentrations of concern for human and wildlife health. Mercury (Hg) pollution in the Arctic environment may worsen as these areas warm and Hg, currently locked in permafrost soils, is remobilized. One of the main concerns is the development of Hg methylation hotspots in the terrestrial environment due to thermokarst formation. The extent to which net methylation of Hg is enhanced upon thaw is, however, largely unknown. Here, we have studied the formation of Hg methylation hotspots using existing thaw gradients at five Fennoscandian permafrost peatland sites. Total Hg (HgT) and MeHg concentrations were analyzed in 178 soil samples from 14 peat cores. We observed 10 times higher concentrations of MeHg and 13 times higher %MeHg in the collapse fen (representing thawed conditions) as compared to the peat plateau (representing frozen conditions). This suggests significantly greater net methylation of Hg when thermokarst wetlands are formed. In addition, we report HgT to soil organic carbon ratios representative of Fennoscandian permafrost peatlands (median and interquartile range of 0.09 ± 0.07 μg HgT g^–1^ C) that are of value for future estimates of circumpolar HgT stocks.

## Introduction

For thousands of years, carbon (C) and mercury (Hg) have been sequestered and “locked” in Arctic and subarctic permafrost soils. Today, northern permafrost peatlands cover 1.7 ± 0.5 M km^2^ and store an estimated 190 ± 70 Pg C and ∼560 to ∼1700 Gg of Hg in the top 3 m.^1−5^ As a consequence of climate change-induced warming, a proportion of these C and Hg stocks is now at risk of remobilization into modern biogeochemical cycles. In circum-Arctic areas with discontinuous permafrost, increases in ground temperature have already been recorded (0.20 ± 0.10 °C from 2007 to 2016).^6^ Warming of permafrost soils across the circumpolar north is expected to continue at an accelerated rate.^7^

Mercury is a global pollutant, subject to long-range transport in the atmosphere in its reduced form (elemental Hg, Hg^0^). While most of the Hg in soil is stored as inorganic divalent Hg (Hg^II^), its methylated form (MeHg) accumulates in aquatic food webs and poses the main risk to human and wildlife health. Warmer soil temperatures in permafrost areas can perturb the biogeochemical cycle of Hg in multiple ways. On local to regional scales, the formation of wet post-thaw environments conducive to microbial conversion of Hg^II^ to MeHg is of major concern, as it could increase the likelihood of enhanced Hg exposure for Arctic residents who are already subject to notably high concentrations of MeHg due to consumption of high trophic level fish and marine mammals.^8^

Thawing permafrost results in the degradation of permafrost peatland landscape features such as palsas (domed peat mounds of segregated deposits of ice and frozen peat) and peat plateaus (flatter expanses of raised, ice-rich peat).^9,10^ As a result of permafrost thaw, the uplifted ground may subside into thermokarst wetland features such as collapse scar fens or thaw ponds.^11−14^ These features typically result in anoxic environments that support communities of anaerobic microorganisms known to methylate Hg, including sulfate- and iron-reducing bacteria and methanogens.^15−17^ High rates of Hg methylation have, for example, been documented in thaw ponds in the Canadian High Arctic by Lehnherr et al.^18,19^ Other studies have also found increased concentrations of MeHg dissolved in pond waters and thermokarst soil pore water.^20,21^ Although most, but not all, of these studies support the formation of Hg methylation hotspots upon permafrost thaw, the literature is currently limited with respect to the approaches applied as well as their geographical coverage. Our understanding of the extent to which such Hg methylation hotspots are formed, and the attendant risk of Hg pollution in downstream systems, is thus poor.

Permafrost peatlands cover extensive areas in subarctic Fennoscandia.^22^ In recent decades, substantial loss of Fennoscandian permafrost, including palsas and peat plateaus, has been documented.^6,11,13,23^ Further deepening of the active layer and the degradation and collapse of palsas and peat plateaus is likely, given the already relatively warm soil temperatures of the area (close to 0 °C) and the ongoing warming of the Arctic.^11,23−25^ Here, we have studied the formation of Hg-methylation hotspots using existing thaw gradients at five Fennoscandian sites. This was done by quantifying total Hg (HgT) and MeHg, from which % MeHg (used as a proxy for net methylation of Hg) was calculated, along with percent soil organic carbon (% SOC), percent nitrogen (% N), and the isotopic signature of carbon (δ^13^C) and of nitrogen (δ^15^N) in 14 peat cores covering intact peat plateaus and collapse scar fens (hereafter referred to as “collapse fens”) which represent frozen and thawed environments, respectively, and distant fens with no recent history of permafrost included as a reference. In addition to Hg methylation, we present and discuss HgT to SOC ratios of value for future estimates of circumpolar permafrost soil HgT stocks.

## Materials and Methods

### Study Sites

**Figure 1:**
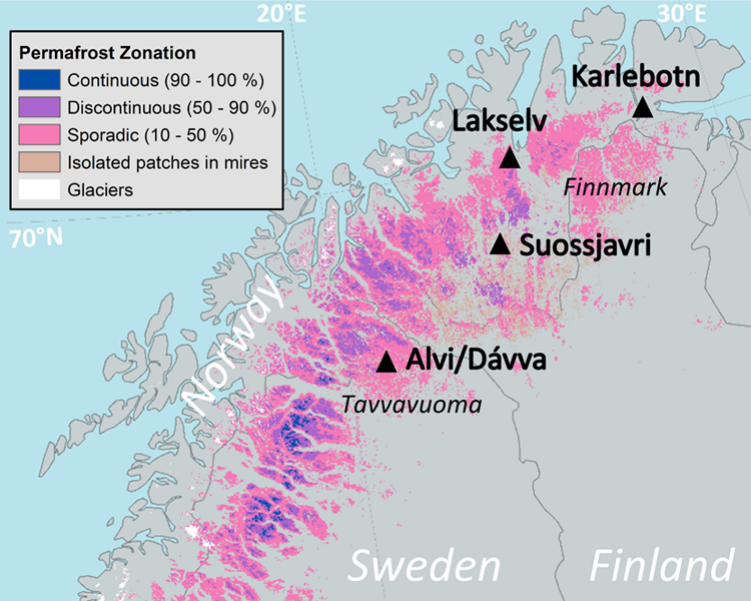
Locations of the studied in situ thaw gradients, comprising two coastal sites (Karlebotn and Lakselv) and one inland site (Suossjavri) from Finnmark, Norway, and two inland sites (Alvi and Dávva) from the Tavvavuoma mire complex, Sweden. The map showing permafrost zones is reprinted with permission from Gisnås et al.^26^

The five study sites, Alvi, Dávva, Lakselv, Karlebotn, and Suossjavri (Figure 1), represent permafrost peatlands typical of subarctic Fennoscandia and consequently share many characteristics. These include landscape features, a general history of post-glacial peatland development followed by permafrost aggradation, mean precipitation (for the continental sites, though this differs between coastal and inland locations), mean annual ground temperature, and characteristic vegetation. Further information on the study sites is available in the Supporting Information.

### Field Sampling

Legacy peat plateau (active layer and permafrost) samples taken in Tavvavuoma in August 2012 (the study sites Alvi and Dávva) and in Finnmark in September 2016 (Karlebotn, Lakselv, and Suossjavri) were combined with field sampling of collapse and distant fens at all sites in August and September 2019. The five collapse fen cores were taken from thermokarst formations adjacent to the sampled plateaus. The other four cores were sampled in the so-called “distant” fens which, on the basis of mapping using historical aerial photographs,^11,13^ were thought unlikely to have had permafrost since the 1950s at least and thus represent the control (non-permafrost affected peatland).

All peat plateau sampling sites were located at a distance from thinner and/or potentially more degraded plateau edges (Figure S1).^27,28^ For the Alvi and Dávva sites, active layer samples were collected by hammering a steel pipe into the peat, pressing out the core, and subdividing the material into 2.5 cm increments in the field.^28^ At the Finnmark sites, blocks of peat were cut out using a handsaw and subdivided into 2.5 segments at the Stockholm University labs.^27^ To core the permafrost at all sites, steel pipes were hammered into the peat plateau.^27,28^ The sample material was carefully extruded, subdivided into 2.5 cm increments, and bagged in the field. All active layer and permafrost samples were kept frozen following the return from the field.

In order to sample recent collapse fens (i.e., those formed no more than 5 - 10 years ago), these cores were taken at the edge of the fen within 0.5 m of the actively eroding peat plateau edge. As that maximum erosion rates of >7 m/decade have been recorded at the Tavvavuoma mire complex along degrading peat plateau margins abutting thermokarst lakes, where wave activity likely enhances erosion, we estimate the collapse fens to be ∼1 to 5 years in age.^13^ Given the similarities between the study sites, the Finnmark collapse fens are assumed to have similar erosion rates and thus ages. Field sampling began with visual identification of fens adjacent to currently thawing permafrost and subsiding peat near the peat plateau sampling location. Where signs of active erosion such as cracks in the peat or block erosion were not readily apparent, nascent collapse fens were identified by the presence of submerged and dying specimens of dwarf shrubs (e.g., *Betula nana*) that are common to dry bogs/raised plateaus but do not thrive in waterlogged conditions (Figure S2). The distant fens were chosen based on the topography of each site and were taken at least 10 m from the peat plateau edge (Figure S3). All fen cores were collected using a half-cylinder Russian peat/sediment corer measuring 5 by 50 cm with a total volume of 490 cm^3^. All cores were transferred to clean plastic half-pipes measuring 50 cm in length and wrapped securely in clean plastic sheeting. The cores were kept at ambient air temperature (around 17 to 19 °C) during transport back to the lab (within 2 weeks) where they were subsampled into 2.5 cm increments every 10 cm (e.g., 2.5–5, 12.5–15, and 22.5–25 cm). Previous in situ measurements of soil temperatures at Tavvavuoma suggest the daily mean ground temperature in the upper 50 cm to be 7–8 °C in the distant fen and 2–8 °C in the peat plateau (Table S1).^25^ Similar ground temperatures are assumed at the collapse fens as well as at the other sites, as these have a similar climate. Although these cores were transported at temperatures above the in situ temperatures, as discussed in the Supporting Information, the increased temperatures are unlikely to explain any of the trends observed.

### Sample Preparation and Analysis

In total, 178 samples from 14 peat cores (representing the landscape feature classes peat plateau, collapse fen, and distant fen) were analyzed according to routine procedures as described in the Supporting Information for % SOC, % N, δ^13^C, δ^15^N, and HgT and MeHg concentrations. Further information on sample preparation and analysis is available in the Supporting Information. All concentrations are reported as per the dry weight of soil, unless otherwise stated.

### Statistical Analysis

The data-distribution of HgT and MeHg concentrations and % MeHg was assessed using the Shapiro–Wilk normality test as well as visual inspection of density plots and *Q*–*Q* plots.^29^ The tested parameters were deemed to be log–normal and adjusted accordingly using a log10(*x* + 1) transformation followed by interpolation of the data prior to statistical analysis; δ^13^C was log10(*x* + 40) and δ^15^N log10(*x* + 2) transformed. A one-way ANOVA to determine whether the data varied as a result of site-specific factors was then carried out to test for statistically significant differences between the five study sites. Further one-way ANOVAs were subsequently performed on the log-transformed, non-normalized data to determine if and for which core types HgT, MeHg, % MeHg, % SOC, % N, C/N, δ^13^C, and δ^15^N were higher or lower in relation to the other core classes for the top 50 cm, top 1 m, and peat (organic soil)-only portions of the cores. Soil with SOC of 17% or higher was classified as organic, while horizons with SOC below 17% was classified as mineral soil.^30^ To reduce the likelihood of a type I error resulting from multiple one-way ANOVAs, two-way ANOVAs were also conducted to investigate differences in HgT, MeHg, % MeHg, % SOC, % N, C/N, δ^13^C, and δ^15^N with depth (0–50 cm compared to 50 cm to max peat depth) and with core class. Tukey’s pairwise post-hoc test was combined with all ANOVAs to facilitate the interpretation of results. Correlation analysis was conducted using Pearson’s correlation test with correlation coefficient, *r*, and correlation probability, *p*, reported. For all statistical tests, significance was indicated by *p* (probability value) < 0.05. The one-way and two-way ANOVAs and Tukey’s pairwise post-hoc were carried out in PAST^31^ while the boxplot and all correlation testing was done using JMP Pro (version 15.0.0). Unless otherwise stated, all data are presented as ranges or weighted median ± interquartile range (IQR).

## Results and Discussion

### Concentrations of HgT and MeHg

Across the five Fennoscandian sites and 14 peat cores, we observe HgT concentrations ranging from 13 to 210 ng g^–1^ in the organic soil and from 1.1 to 15 ng g^–1^ in the mineral soil layer (Table S2). These observations are similar to previously reported concentrations of HgT from permafrost peatland sites in Abisko, relatively close to our more southerly sites (∼12 ng Hg g^–1^ in the organic-poor portions of the peat cores and near-surface peaks ranging from 95 to 330 ng Hg g^–1^).^32^ The median (± IQR) HgT concentration is 35 ± 30 and 4.2 ± 6.3 ng g^–1^ in the organic and mineral soil, respectively. For MeHg, the concentration ranges from 0.012 to 28 ng g^–1^ (median ± IQR of 0.51 ± 1.0 ng g^–1^) in the organic peat soil and from 0.0041 to 0.21 (median ± IQR of 0.030 ± 0.041) ng g^–1^ in the underlying mineral layer (Table S2). MeHg concentration data from other Fennoscandian permafrost soils are currently lacking. Observed concentrations of MeHg are, however, comparable to the limited number of studies available from other Arctic soils, for example, from northern Alaska (<0.1 to 3.4 g^–1^, *n* = 5),^3^ from northern Canadian wetlands on Cornwallis Island (0.065 ± 0.074, *n* = 75)^33^ and Truelove Lowland (0.35 ± 0.04, *n* = 35 to 3.1 ± 0.52, *n* = 35).^34^ Below, we discuss the depth distribution of HgT, *R*_HgTC_ (HgT normalized to organic carbon content), MeHg, and % MeHg as well as the geochemical properties of % SOC, % N, C/N, δ^13^C, and δ^15^N within cores, between sites and between core classes (peat plateau, collapse fen, and distant fen).

### Distribution of HgT

**Figure 2:**
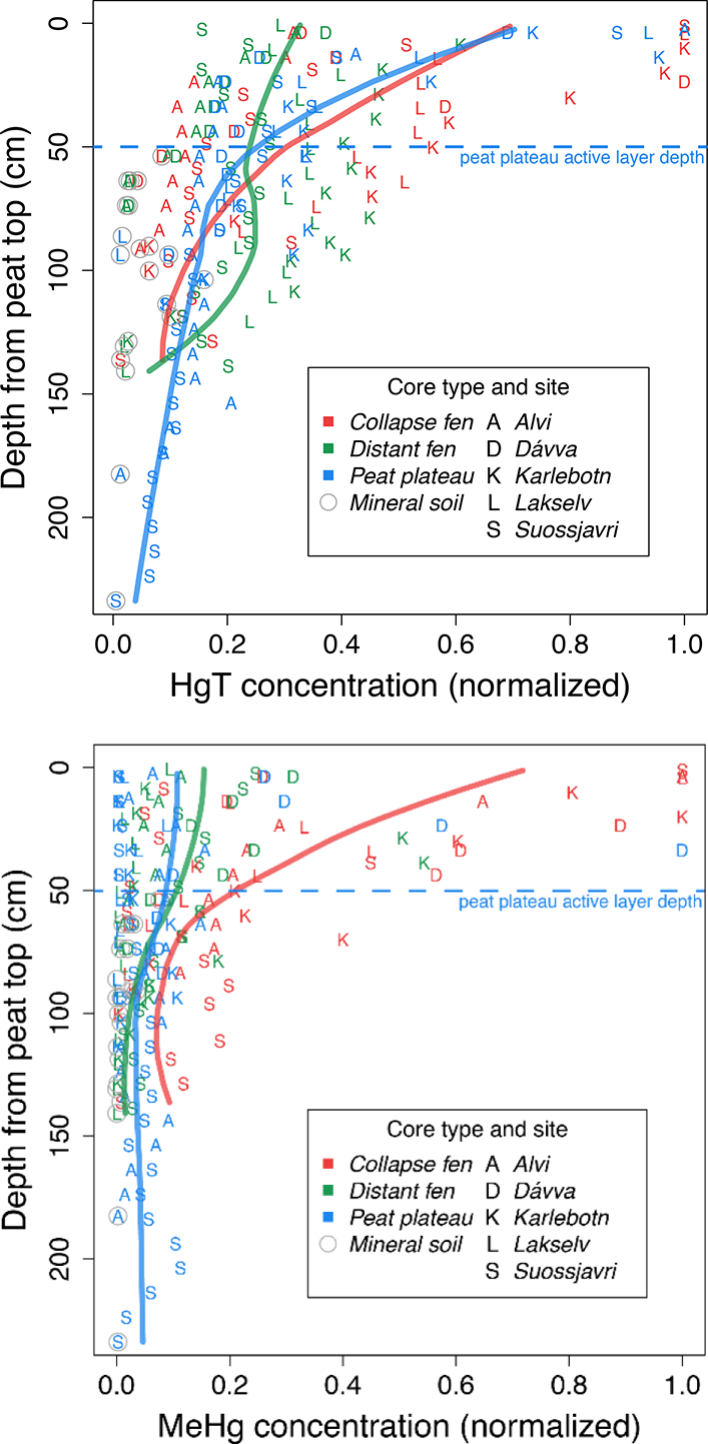
Depth-distribution of HgT (top) and MeHg (bottom) concentrations (normalized to the maximum value of each site) for each core class, compiled across all sites, with local regression (LOESS). Active layer depth for the peat plateaus was around 50–55 cm.^27,28^

Within the cores, a general trend of decreasing HgT concentration with depth, with the lowest concentrations in the mineral soil layer, was observed (Figures 2 and S4, correlation test for HgT vs depth: *r* = −0.56, *p* < 0.0001). This trend is more pronounced in peat plateau and collapse fen cores in comparison to distant fen cores. Between sites and between core classes, no significant difference in HgT concentrations in the top 50 cm, the top 1 m, or the peat-only core portions was observed (Tukey’s post-hoc following one-way ANOVA, *p* > 0.05), with one exception (Dávva < Suossjavri, for the peat-only interval; Tukey’s post-hoc following one-way ANOVA, *p* < 0.05, Table S3). The difference in HgT between Dávva and Suossjavri may be due to the fact that, in comparison to the other sites, the Dávva cores were particularly short, while the Suossjavri cores were relatively long (Figure S4).

### Correlation of HgT with Organic Matter Content and Quality

HgT is known to co-cycle strongly, though not exclusively, with organic matter (OM).^35^ Across all sites and core classes, % SOC and % N remain high throughout the peat portion of the cores (median SOC of 44 ± 9.0% and median N of 2.1 ± 0.73%) before dropping as the mineral layer is reached (median of 1.6 ± 5.2% and 0.1 ± 0.29% for SOC and N, respectively) (Figures S5 and S6). When both organic and mineral soil layers are included in the analysis (*n* = 178), HgT also correlates strongly with % SOC (*r* = 0.77, *p* < 0.0001) and % N (*r* = 0.72, *p* < 0.0001), lending support to OM as the main driver of Hg distribution. This correlation is, however, weakened when the organic soil alone is tested (*n* = 159, HgT vs % SOC: *r* = 0.23, *p* < 0.01, HgT vs % N: *r* = 0.18, *p* < 0.05). Furthermore, observed trends of decreasing HgT concentrations with depth in the near-surface peat remain even after normalizing HgT concentrations to % SOC (a ratio hereafter referred to as *R*_HgTC_) (correlation test for HgT vs depth in the organic peat layer: *r* = −0.50, *p* < 0.0001, Figure S7). The vertical profile of Hg in the organic soil portion of the cores does not necessarily reflect historical trends in atmospheric deposition of HgT alone, as HgT may also be mobilized vertically within the soil profile. HgT mobilization in the upper 50 cm of the cores may, for example, be driven by frequent shifts in redox conditions due to fluctuations of the water table or by the relocation of HgT in higher, more surficial soil sections during annual freeze-thaw cycles.^36,37^ Earlier work has demonstrated a negative correlation between *R*_HgTC_ and C/N, wherein older, more degraded soils (indicated by a lower C/N ratio) were associated with higher *R*_HgTC_ values.^38,39^ Obrist et al.^38^ attribute this trend to the longer exposure time these older soils have had for Hg deposition and/or to *R*_HgTC_ increasing as a result of the more rapid depletion of C than Hg during soil remineralization. In our study, we observe a negative correlation between *R*_HgTC_ and C/N (*r* = −0.26, *p* < 0.001) and a positive correlation between both *R*_HgTC_ and δ^13^C (*r* = 0.44, *p* < 0.0001) and *R*_HgTC_ and δ^15^N (*r* = 0.34, *p* < 0.0001). These results support previous findings of higher *R*_HgTC_ values in older and more degraded soils. When analyzing data from the organic soil layer, however, a significant correlation is only present between *R*_HgTC_ and δ^15^N (*r* = 0.46, *p* < 0.0001), while no significant correlation was found between either *R*_HgTC_ and C/N or *R*_HgTC_ and δ^13^C (*r* > 0.05).

Although % SOC explained little of the HgT variability in the organic soil, *R*_HgTC_ nonetheless warrants discussion, as this ratio has recently been used to estimate the soil HgT pool of the Arctic and subarctic permafrost regions.^2−4^ In the first calculated estimate, Schuster et al.^2^ multiplied a median *R*_HgTC_ of 1.6 ± 0.9 μg HgT g^–1^ C, derived from measurements of Alaskan tundra soils, with estimates of C storage in the Arctic.^1,40^ Since then, additional work has demonstrated the need to account for regional differences and soil type in the *R*_HgTC_ used for estimates of pan-Arctic Hg stocks. Olson et al.,^3^ for instance, derived median *R*_HgTC_ values ranging from 0.12 μg HgT g^–1^ C in organic soil (0–30 cm depth) to 0.62 μg HgT g^–1^ C in mineral soil (30–100 cm depth) by combining their data with HgT and SOC data from more than 30 published studies conducted in Arctic tundra and boreal regions. (Note that Olson et al.^3^ report the median organic soil *R*_HgTC_ to be 0.27 μg HgT g^–1^ C, a value Lim et al.^4^ have identified as a typo; the corrected value of 0.12 μg HgT g^–1^ C is thus presented here). Of greater relevance to this study, due to the permafrost peatland nature of the study location, is the median *R*_HgTC_ of 0.13 ± 0.12 μg HgT g^–1^ C for Western Siberian Lowland peat bogs measured recently by Lim et al.^4^ In their Western Siberian peat profiles, *R*_HgTC_ values increased 5- to 10-fold from the organic (SOC >20%) to the mineral (SOC <20%) soil. Lim et al.^4^ further combined their measurements with published data to revise the median *R*_HgTC_ to 0.15 μg HgT g^–1^ C in organic soils and 0.64 μg HgT g^–1^ C in mineral soils for North America and Eurasia.

In Fennoscandian permafrost peatlands, we observe a median *R*_HgTC_ of 0.09 ± 0.08 μg HgT g^–1^ C across the investigated core classes and sites (Table S2). The *R*_HgTC_ was consistent across core classes, ranging from 0.07 ± 0.07 μg HgT g^–1^ C in the peat plateau to 0.10 ± 0.06 μg HgT g^–1^ C in the distant fens and 0.12 ± 0.12 μg HgT g^–1^ C in the collapse fens (Table S4). The median *R*_HgTC_ for all mineral soil samples at the base of peat deposits was higher, at 0.26 ± 0.20 μg HgT g^–1^ C, than the median *R*_HgTC_ for organic soils, at 0.09 ± 0.07 μg HgT g^–1^ C (Table S2). These findings are in line with the abovementioned work suggesting lower *R*_HgTC_ values in organic as compared to mineral soils, as well as with lower whole-core median values than those applied by Schuster et al.^2−4^ The disparity in median whole-core *R*_HgTC_ values between this study and that of Schuster et al.^2^ may be due largely to the sampling location and the character of the sampled deposit. As the cores of this study represent a permafrost peatland environment, % SOC is higher (median and IQR 44 ± 10%) than for Schuster et al.’s Alaskan tundra soils, which are much more mineral-rich (median SOC of 3% and IQR 1.7–8.7%) (see Figure S8 and Table S2).^2,4^ Given the lower ratio measured in organic soils, this study’s median *R*_HgTC_ may be lower overall than that of Schuster et al.^2^ because organic soils accounted for a majority of samples (159 out of a total of 178). We note, however, that the mineral soil samples analyzed here are from just below the basal peat and are thus unlikely to be representative for near-surface mineral permafrost soils. Comparisons between the measured *R*_HgTC_ of this study and Lim et al.,^4^ both of which were conducted on organic-rich peatland soils, are therefore more appropriate and pertinent to discussions of *R*_HgTC_ and upscaling of the permafrost soil Hg pool. It is also worth noting that the permafrost in these peatlands is epigenetic, while much of the broader permafrost C pool is characterized by syngenetic permafrost conditions which are likely more conducive to Hg preservation in the soil. Finally, there are likely also large regional differences in natural Hg sources. Lim et al.^4^ point out that HgT concentrations in the area Schuster et al.^2^ and Olson et al.^3^ sampled, along the Dalton Highway in Alaska, tend to be unusually high. This may be due to particularly high geogenic contributions of HgT from weathering bedrock or, perhaps, to a greater loss of C from mineral than organic soils in that region due to mineralization of C.

Schuster et al.^2^ have suggested that 863 ± 501 and 793 ± 461 Gg of HgT may be stored in the active layer and in the perennially frozen substrate, respectively, such that the top 3 m of northern permafrost soils contain 1656 ± 962 Gg HgT in total. Lim et al.^4^ have revised this estimate to 408 Gg (range of 319–584 Gg)^3^ of Hg stored in the top 1 m, with 557 Gg (IQR: 371–699 Gg) of Hg stored in the top 3 m of northern permafrost soils. The range of *R*_HgTC_ reported across systems and soil types (0.13 ± 0.12 μg HgT g^–1^ C for Western Siberian Lowland peatlands to 1.6 ± 0.9 μg HgT g^–1^ C for Alaskan tundra soils) underscores the need to incorporate the empirical relationship between HgT and % SOC for minerogenic and organic soils separately in order to derive *R*_HgTC_ values that better represent the heterogeneity of Arctic and subarctic permafrost regions. With a median *R*_HgTC_ of 0.09 ± 0.07 μg HgT g^–1^ C across investigated core classes and sites (14 cores, *n* = 178), our observations support the revised stock estimate of Lim et al.^4^ The lack of statistical differences between the five sites (one-way ANOVA, *p* > 0.05, Table S3), and between core classes (*p* > 0.05 for both one and two-way ANOVA, Tables S5 and S6), suggests the calculated *R*_HgTC_ to be representative for Fennoscandian permafrost peatlands and of high value for further stock estimates of Hg in the circumpolar north.^2−4^

### Distribution of MeHg and % MeHg

**Figure 3:**
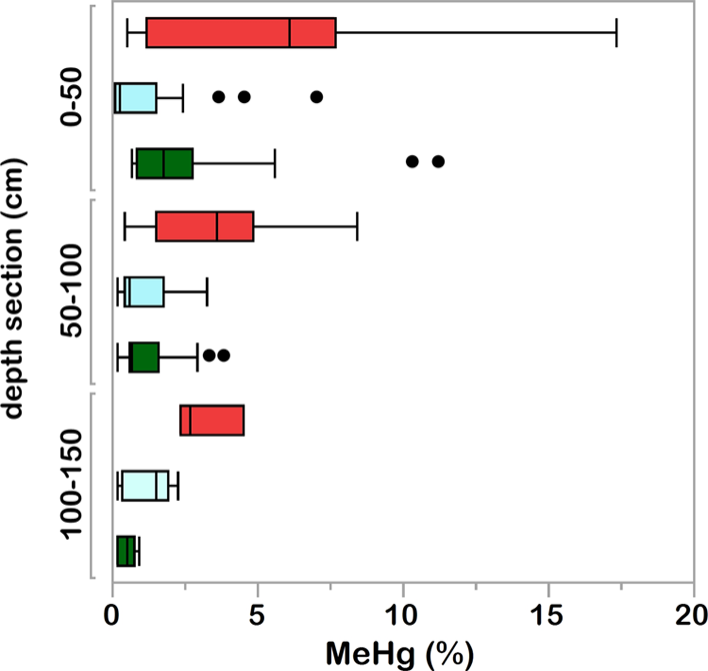
% MeHg in the top 0–50, 50–100, and 100–150 cm sections of the peat core collected from collapse fen (first uppermost red bar), peat plateau (second uppermost light blue bar), and distant fen cores (third uppermost green bar). Boxes indicate the upper and lower (75th and 25th) percentiles, horizontal line the median, error bars the inter-quartile range, and circles the outliers.

Concentrations of MeHg and % MeHg typically reach their maxima in the upper portion of the near-surface peat layer (Figures 2, 3 and S9). This pattern is consistent with observations from other soil systems, including soils from the Tibetan Plateau.^41^ A significant, but weak correlation for MeHg and % MeHg with depth was also observed (*r* = −0.27, *p* < 0.001 for MeHg and *r* = −0.20, *p* < 0.01 for % MeHg). No significant difference in MeHg concentrations or % MeHg was observed between the five Fennoscandian sites for any of the tested portions (one-way ANOVA, *p* > 0.05 for the top 50 cm, top 1 m, and the entire organic soil section, Table S3). Concentrations of MeHg in the upper 50 cm range from 0.17 ± 0.55 ng g^–1^ in the peat plateaus to 0.78 ± 1.2 ng g^–1^ in the distant fens and 1.8 ± 4.7 ng g^–1^ in the collapse fens. These concentrations correspond to upper 50 cm % MeHg values of 0.48 ± 1.7%, 1.7 ± 2.4%, and 6.1 ± 6.5% in the peat plateau, distant fen, and collapse fen classes, respectively. The 10 times higher MeHg concentration and 13 times higher % MeHg in the top 50 cm of the thawed collapse fen compared to the intact peat plateau suggests significantly higher net methylation of Hg when thermokarst wetlands are formed (Tukey’s post-hoc following one-way ANOVA, *p* < 0.01 for MeHg and *p* < 0.05 for % MeHg, Table S5 and Tukey’s post-hoc following two-way ANOVA, *p* < 0.05 for MeHg and % MeHg, Table S6). Higher MeHg and % MeHg in the collapse fen compared to the peat plateau is also supported when MeHg and % MeHg are analyzed in relation to depth (Tables S5 and S6). Median concentrations of % MeHg in the top 50 cm are 3.6 times higher in the collapse fen than in the distant fen. Higher % MeHg in the collapse fen compared to the distant fen is also supported by Tukey’s post-hoc results following the two-way ANOVA (Table S6) and by Tukey’s post-hoc following one-way ANOVA for the peat-only section but not for the top 50 cm or the top 1 m (Table S5). When MeHg and % MeHg as a function of both depth and core class are investigated, no difference in MeHg concentration or % MeHg is apparent between the shallower (<50 cm) and deeper (>50 cm to max peat depth) peat (two-way ANOVA, p > 0.05, Table S6).

### Increased Net Methylation of Hg upon Thaw

There are a number of processes that can explain increased Hg-methylation following permafrost thaw and the creation of collapse scar fens or thaw ponds. The water table is known to be the main driver of redox conditions in peat soils.^42^ As the peat surface of the collapse and distance fens was submerged well below the water table, anoxic conditions within centimeters of the peat surface are expected. As mentioned previously, such conditions may enhance MeHg production by supporting communities of anaerobic microorganisms known to methylate Hg.^15−17^ Fahnestock et al.,^21^ for example, found a higher abundance and more diverse set of potential Hg methylators (including sulfate- and iron-reducing bacteria and methanogens) in the thawed portion of a subarctic thaw gradient geographically close to our investigated sites. Notably, a high abundance of the *hgcAB* genes, mostly found in methanogens and *Firmicutes*, in thawing permafrost soils was also observed in a global screening of microbial metagenomes from a variety of environments.^17^ These field observations align with observations from a laboratory study in which permafrost soils from Alaska were incubated and a close correlation between the production of MeHg and the production of CH_4_ and reduction of iron was shown (suggesting the involvement of methanogens and/or iron-reducing bacteria^16^).^43^

Although we expect comparable redox conditions at the collapse and distant fens, statistically higher % MeHg (in comparison to the peat plateau core class) was only observed in the collapse fen class, suggesting that redox conditions alone do not drive the higher % MeHg observed. In addition to promoting the diversity and abundance of potential Hg methylators, thermokarst wetland formation may enhance the activity of these microorganisms as “fresh” OM becomes available for remineralization. In the surface waters of eastern Canadian Arctic ponds and lakes, for instance, MacMillan et al.^20^ found a strong correlation between levels of MeHg and inputs of dissolved organic carbon and nutrients as well as between MeHg and variables associated with microbial activity. In the aforementioned laboratory study, Yang et al.^43^ also observed high MeHg production to coincide with a rapid loss of the water-soluble labile SOC pool and addition of a labile organic compound (glucose) to enhance Hg methylation. As the collapse fen cores included in our study were sampled close to the actively degrading peat plateau edge, we assume these fens to be fairly young (∼1–5 years old) in comparison to the non-permafrost impacted distant fens. Higher % MeHg at these sites could thus be due to the fact that the collapse fens were flooded more recently and still contain enough fresh OM to support greater microbial activity. The temporal aspect of enhanced MeHg production upon flooding has previously been explored for other terrestrial environments where reservoirs have been constructed, typically through monitoring of MeHg in biota from the reservoirs. These studies indicate that MeHg production peaks within a few years, up to a decade,^44^ thus suggesting that age of the waterlogged feature could be an important factor controlling MeHg production upon thermokarst formation. Over what length of time we could expect enhanced MeHg production in comparably colder permafrost systems is a question in need of further study.

Microbial activity alone may not be responsible for the observed differences between the collapse and distant fens. As the distant fens, in general, were assumed to be more hydrologically connected than the collapse fens, increased export of MeHg over longer timespans could have contributed to the lower bulk MeHg and % MeHg of the distant fens. Increased levels of MeHg at the collapse fens may also have been influenced by the greater availability of Hg^II^ for methylation due to remobilization of Hg from the soil matrix as OM is degraded and to increasing concentrations of low-molecular-weight dissolved organic molecules upon thaw which may form bioavailable Hg-DOM complexes.^45,46^

The tight coupling between MeHg production and OM remineralization, as demonstrated by the group of potential Hg methylators present^17,21^ and the correlation between MeHg and CH_4_ and/or CO_2_ production,^20,43^ suggests that understanding carbon cycling in thawing permafrost systems could help elucidate the Hg cycle. The C/N ratio has, for example, been put forth as a potential proxy for OC remineralization availability^47^ and, alongside other geochemical properties such as the humification proxies δ^13^C and δ^15^N, could thus be of interest in future estimates of MeHg production. Our results do not, however, support the use of C/N, δ^13^C, or δ^15^N as proxies for MeHg formation as no significant differences were observed between any of these variables and core class despite the fact that % MeHg differed significantly with core class (Tables S5 and S6) and % MeHg did not correlate with C/N, δ^13^C, or δ^15^N (*p* > 0.05). It is nevertheless possible that these proxies may be of use in predicting MeHg production on larger scales or between more heterogeneous systems.

Our data suggest net methylation in collapse fens (top 50 cm), representing relatively recently thawed conditions, to be an order of magnitude higher in comparison to net methylation in the active layer (top 50 cm) of intact peat plateaus. These observations reveal a risk of increased MeHg exposure to human and wildlife upon permafrost thaw and support earlier work demonstrating the creation of Hg methylation hotspots.^19−21,43^ Lehnherr et al.,^19^ for instance, observed potential methylation rates of Hg^II^ in two thaw ponds which resemble the rates observed in more productive systems south of the permafrost region, such as temperate wetlands and lakes. In the Stordalen mire, close to our more southerly sites, Fahnestock et al.^21^ found higher concentrations of MeHg in soil pore water in recently thawed soils along a thaw gradient. Along with increased levels of dissolved MeHg, the authors also observed a higher abundance of potential Hg methylators. High levels of MeHg have also been measured in the bottom waters of thaw ponds in eastern Canada.^20^ Previous approaches applied are, however, limited in their ability to assess the degree to which net methylation changes upon thawing of the permafrost soil.^48^ Quantification of potential Hg^II^ methylation rates is a powerful approach allowing us to compare methylation rates between systems and to identify possible drivers. Despite this, these rates are known to be of limited use when assessing the net methylation of Hg in an environmental system.^48^ First of all, the pool of MeHg is not only controlled by Hg^II^ methylation rates but also by demethylation processes and the availability of substrates (Hg^II^ and electron donors/acceptors). Oiffer and Siciliano^34^ found Arctic soils in the Truelove Lowland act as a sink for MeHg during the summer months, for instance.^34^ Lehnherr et al.^19^ further determined that photochemical demethylation degrades a substantial portion of the methylated Hg in Canadian thaw ponds. Second, tracers used for the determination of potential methylation rates are known to have a higher availability for methylation in comparison to ambient Hg^II^, unless the chemical speciation of the ambient Hg^II^ pool is carefully mimicked.^49^ Estimated rates are thus typically an order of magnitude higher than the methylation rates of ambient Hg (depending on the chemical speciation of the two pools).^49,50^ Linking levels of dissolved MeHg in ponds or pore water to net Hg methylation also warrants some caution, unless differences in the solubility of MeHg between systems are accounted for.^51^ It should nonetheless be noted that such measurements are important as they may, to a greater extent than bulk measurements, reflect the amount of MeHg available for transport to and subsequent accumulation in downstream systems. To our knowledge, we provide the first estimate of net MeHg formation upon thaw through quantification of bulk % MeHg in in situ soil thaw gradients. The importance of such data in assessing current and future risks of Hg in the Arctic is further heightened by past work where terrestrially derived MeHg has been shown to greatly contribute to the pool of Hg accumulating in downstream food webs, even in systems where its contribution to the abiotic compartment is minor.^52,53^

## Supporting Information Available

Photos from study sites, geochemical analysis, analysis of HgT and MeHg, ground temperatures in Tavvavuoma, summary statistics (HgT, MeHg, % MeHg, *R*_HgTC_, and ancillary parameters in peat and mineral layer; peat soil classes and with peat depth), one-way ANOVA and Tukey’s post-hoc (comparing HgT, MeHg, % MeHg, and *R*_HgTC_ between sites, comparing HgT, MeHg, % MeHg, and *R*_HgTC_ and ancillary parameters between core classes), two-way ANOVA and Tukey’s post-hoc (comparing HgT, MeHg, % MeHg, and *R*_HgTC_ and ancillary parameters between core classes and with depth), depth-distribution of HgT conc. by site, depth-distribution of % SOC by core class and by site, depth-distribution of all parameters for each site, depth-distribution of HgT concentration normalized to C by core class and by site, HgT as a function of % SOC (*R*_HgTC_), depth distribution of % MeHg across all sites and core classes, and discussion on the potential effect of sampling transportation (PDF).
