# Supplementary material for: Permafrost Thaw Increases Methylmercury Formation in Subarctic Fennoscandia
Source: Environ Sci Technol. 2021 Apr 27;55(10):6710–7. doi: 10.1021/acs.est.0c04108 (PMC8277125; doi:10.1021/acs.est.0c04108)
Supplement: Supplementary file 1 — es0c04108_si_001.pdf [file es0c04108_si_001.pdf]

Supporting Information

Permafrost thaw increases methylmercury formation in subarctic Fennoscandia

Brittany Tarbier, Gustaf Hugelius, A. Britta K. Sannel, Carluvy Baptista-Salazar, Sofi Jonsson

Corresponding Author: Sofi Jonsson, sofi.jonsson@aces.su.se

Index

Supplementary Information for Material and Methods **S1**
Study Sites ***S1***
Figure S1. Karlebotn peat plateau coring site *S2*
Figure S2. Karlebotn collapse fen coring site *S3*
Figure S3. Tavvavuoma distant fen coring site *S3*

Sample preparation and analysis ***S4***
Geochemical analysis *S4*
Analysis of HgT *S4*
Analysis of MeHg *S4*

Supplementary Information for Results and Discussion **S6**
Table S1. Ground temperatures, Tavvavuoma *S6*
Table S2. Summary statistics (peat and mineral layer) *S6*
Table S3. One-way ANOVA and Tukey's post-hoc, sites *S7*
Table S4. Summary statistics for the peat soil classes and peat depth *S8*
Table S5. One-way ANOVA and Tukey's post-hoc, core classes *S11*
Table S6. Two-way ANOVA and Tukey's post-hoc, core classes and depth *S12*
Figure S4. Depth-distribution of HgT conc. by site *S13*
Figure S5. Depth-distribution of %SOC by core class and by site *S14*
Figure S6. Depth-distribution of all parameters for each site *S15*
Figure S7. Depth-distribution of HgT conc. normalized to C by core class and by site *S20*
Figure S8. HgT as a function of %SOC (R_{HgTC}) *S21*
Figure S9. Depth-distribution of %MeHg across all sites and core classes *S21*
Discussion – potential effect of sampling transportation *S22*

References **S23**

Supplementary Information for Material and Methods

Study sites. Alvi (68°27’51”N, 20°54’05”E) and Dávva (68°27’51”N, 20°54’26”E) are located at an elevation of ~555 m.a.s.l. in Tavvavuoma, an expansive permafrost peatland located in the sporadic permafrost zone of northernmost Sweden.[1–3] The Tavvavuoma peat complex is underlain with sandy glacio-fluvial and glacio-lacustrine deposits and contains palsas and peat plateaus, small thermokarst fens and ponds, streams, lakes, wetlands, with low hills to the west and to the east, the larger waterbody, Lake Dávvajavri.[4,5] Peatland inception most likely occurred at Alvi around 9,700 cal. yr BP and 6,000 cal. yr BP at Dávva. Permafrost development at Tavvavuoma generally is dated to around 600 – 100 cal. yr BP.[1]

Lakselv (70°7’15”N, 24°59’47”E, 50 m.a.s.l.), Karlebotn (70°7'14”N, 28°28’30”E, 26 m.a.s.l.) and Suossjavri (69°23’2”N, 24°15’27”E, ~337 m.a.s.l.) are located in the county of Finnmark, in northernmost Norway. In this region of discontinuous and sporadic permafrost palsas and peat plateaus cover ~110 km^2.[3,6] Lakselv and Karlebotn are coastal sites while Suossjavri is located further inland[6,7]. In Finnmark, peatland inception likely commenced around 9,800 cal. yr BP at Suossjavri, ~5,150 cal. yr BP at Karlebotn, and 6,150 cal. yr BP at Lakselv [7]. Initiation of permafrost has been estimated at ~950 cal. yr BP in Karlebotn, around 150 cal. yr BP in Lakselv, and ~100 cal. yr BP in Suossjavri.[7]

The mean annual ground temperature at all study sites is just below freezing, with an approximate late season thaw depth of 55 to 60 cm in Tavvavuoma and 40 to 70 cm in Finnmark[7–9]. In Tavvavuoma the mean annual precipitation for the period 2006 – 2013 was 461 mm/yr (at the nearest meteorological station in Naimakka, ~35 km northeast of Tavvavuoma)[8] while the mean annual precipitation for the Finnmark sites is below 500 mm for the inland locales and above 1000 mm at the coast.[10]

Across these sites, the principal vegetation of the relatively dry peat plateau surfaces consists of dwarf shrubs (*Empetrum nigrum* ssp. *hermaphroditum*, *Betula nana*, *Vaccinium uligunosum* ssp. *uligunosum*, *V. vitis-idaea*, *V. microcarpum*, *Andromeda polifolia*), *Rubus chamaemorus*, lichens, and mosses (e.g. *Polytrichum juniperinum*, *Dicranum elongatum*).[1,7] At the lake edges and in the fens both adjacent to and at a distance from the degrading permafrost plateaus, hydrophytic cotton grasses (*Eriophorum* spp.), sedges (*Carex* spp.) and *Sphagnum* species dominate.

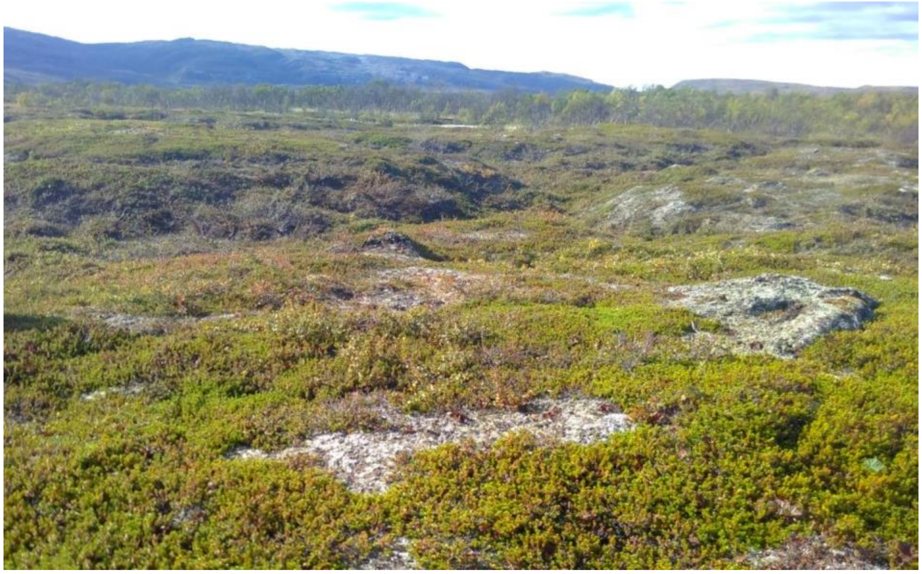

Figure S1. The Karlebotn peat plateau coring site with vegetation typical of a permafrost-raised plateau. In the middle-distance, raised ridges of peat are interspersed with small sunken “valleys” formed through ground subsidence. Photo: Marit Hichens-Bergström.

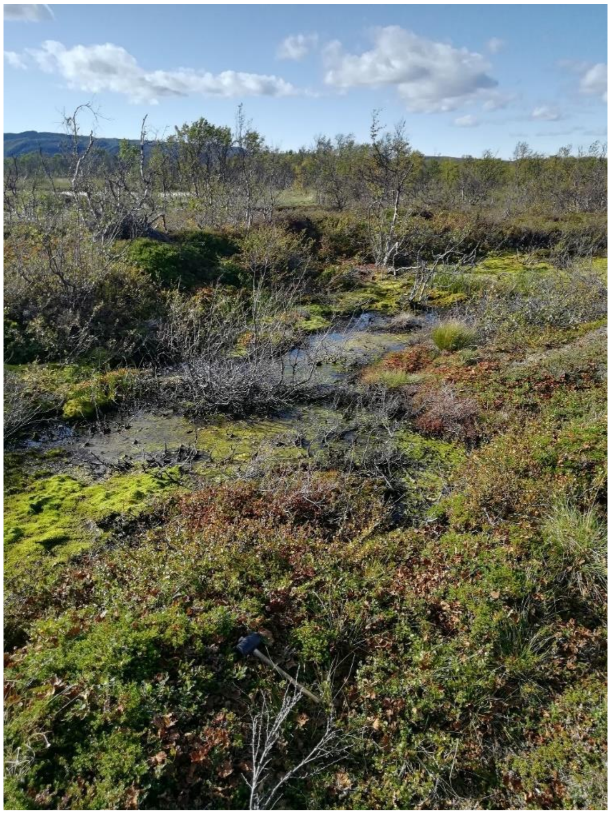

Figure S2. Karlebotn collapse fen coring site. The presence of dying shrubs like dwarf birch (*Betula nana*) suggests recent collapse of this peat plateau and formation of a waterlogged fen. Photo: Brittany Tarbier.

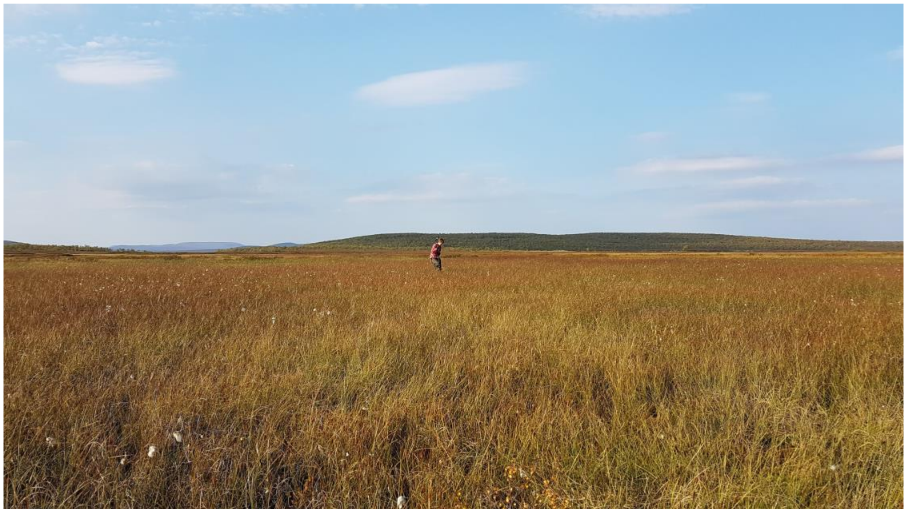

Figure S3. Sampling of the Tavvavuoma distant fen. Typical fen vegetation included cotton grasses (*Eriophorum* spp.), sedges (*Carex* spp.), and mosses (*Sphagnum* spp.). Photo: Freya Sykes.

Sample preparation and analysis. All samples were freeze-dried in a Heto Drywinner 6.55 freeze dryer then homogenized to a fine powder with an agate mortar and/or a scientific-grade IKA A11 Basic Mill. Thorough cleaning using ethanol was conducted between each sample to prevent contamination.

Geochemical analysis. Biogeochemical analysis of %SOC, %N, $\delta^{13}C$, and $\delta^{15}N$ was carried out at the UC Davis Stable Isotope Facility. In order to test for the prevalence of inorganic carbon, ~80% of samples were baked in the lab at Stockholm University at 900° for 4 hours following standard loss on ignition protocols[11]. Inorganic carbon was found to contribute insignificantly to the overall soil composition, with values ranging from 0-3.4%, a mean of 0.82% and a standard deviation (SD) of 0.84. As a result, further analysis of inorganic carbon was not conducted and "carbon" is understood to refer to "organic carbon" for purposes of this study. All %SOC scatterplots were created in R.[12]

Analysis of HgT. Concentrations of HgT was determined through thermal decomposition-atomic absorption spectrophotometry using a Milestone Direct Mercury Analyzer-80 (DMA) following the procedure described in U.S. EPA Method 7473[13]. Approximately 100 (± 5%) mg of homogenized and freeze-dried sample material was weighed out into pre-weighed and cleaned Ni sample boats and analyzed with the DMA. The instrument detection limit is 0.01 ng HgT, with a working range of 0.05 – 600 ng. To minimize the risk of memory effects between samples and ensure DMA-calibration, between every 10 ± 2 samples, an empty sample boat, a boat containing an internal sediment reference standard (previously determined to contain 210 ± 10 ng/g soil), and a boat of replicate sample material was also tested. Throughout HgT testing for this study, the internal standard had a Hg concentration of 201.3 – 221.8 ng/g soil, with a mean of 214.9 ng/g soil, a SD of 3.8, and a relative standard deviation (RSD) of 1.8%. The replicate material was a mineral-rich sample from the Karlebotn collapse fen. For this quality control sample, HgT, in ng/g soil, ranged from 4.3 – 7.6, with a mean of 5.4, a SD of 0.7, and an RSD of 12.2%. All HgT scatterplots were created in R.[12]

Analysis of MeHg. In order to extract MeHg from the soil samples, between ~0.5 – 1 g of material was measured into new 50 mL Falcon tubes and between 20 – 100 µL of an internal standard—an isotopically enriched $Me^{200}Hg$ standard with concentration 1.1 ng/g—added and left to equilibrate for an hour. During a subsequent run, for 11 samples and 3 replicates (each analyzed twice), the amount of tracer was adjusted to between 100 µL and 2.5 mL to improve the analysis, as too little or too much tracer can "mask" the measured MeHg concentration. After equilibration, 10 mL KBr (1.4M), 2 mL $CuSO_4$ (2M) and 10 mL dichloromethane, DCM (CH_2Cl_2) are added to each tube, which is capped and left for 45 minutes. In order to extract MeHg, the samples are rotated at 85 RPM on a sample rotor for 45 minutes, then centrifuged for 5 minutes at 3000 RPM. Glass Pasteur pipettes are used to manually transfer the lower (clear) layer containing DCM and the extracted

MeHg to a new 50 mL Falcon tube. After adding 10 mL of Milli-Q (MQ) water to the pipetted liquid, the DCM is purged in a warm water bath at 45 °C, and the extraction is complete.

MeHg was analyzed using a Tekran® Model 2700 Automated Methylmercury Analysis System connected to an Inductively Coupled Plasma Mass Spectrometer, Thermo–Fisher X-series 2 (ICPMS). Prior to analysis, half the extracted sample was ethylated using sodium tetraethyl-borate (NaTEB) at pH 4.9 (using 225 µl 2M acetate buffer). The resulting data was manually adjusted in Excel to determine the MeHg peak area.

The concentration of MeHg for each sample was subsequently calculated using mass-bias (MB) corrected signals derived through signal deconvolution.[14] Three mass-bias vials, each containing 0.5 ppt ambient Hg ethylated in sodium tetraethyl-borate (NaTEB) at a pH of 4.9 (using 225 µl 2M acetate buffer), were analyzed and MeHg concentration for each sample adjusted by the calculated MB correction factor. Replicate testing was conducted using three peat-rich samples from the topmost sample horizons of the Alvi collapse fen. The mean (in ng MeHg/g soil) and %RSD of the replicates was: 4.30 ± 9.12%, 2.74 ± 10.40%, and 1.20 ± 10.74%. Five blanks, containing only reagents, were tested concurrently with the sampled material to ensure no contamination of MeHg occurred during the extraction process. Certified reference material (ERM-CC580, estuarine sediment) analyzed were on average 110% of the certified value (75 ± 4 ng g^{-1}). All MeHg and %MeHg scatterplots were created in R.[12]

Supplementary Information for Results and Discussion

Table S1. Ground temperatures, Tavvavuoma. Distant fen and peat plateau soil temperatures reported from Tavvavuoma on the 28th of August 2007. Daily mean values are based on 3h interval measurements from Sannel (2020).[15]

Depth (cm)	Fen (distant), daily mean ground temperature (°C)	Peat plateau, daily mean ground temperature (°C)
2	8.41	8.07
25	8.13	4.62
50	6.62	2.00

Table S2. Summary statistics for organic and mineral soil layers, and whole cores across sites (median and interquartile ranges are weighted per core).

	HgT ng/g	MeHg ng/g	MeHg %	R_{HgC} µg Hg/g C	SOC %	N %	C/N	$\delta^{13}C$ ‰	$\delta^{15}N$ ‰
**Organic Soil Layer (SOC > 17%, n=159)**									
Median	**35**	**0.51**	**1.5**	**0.088 (0.085*)**	**44**	**2.1**	**20**	**-28.0**	**0.567**
Interquartile Range	*29*	*1.0*	*3.2*	*0.07 (0.07*)*	*9*	*0.73*	*5.5*	*0.827*	*1.24*
Minimum	13	0.012	0.02	0.031	20	0.87	14	-30.1	-1.35
Maximum	210	28	17	0.43	52	3.3	54	-24.9	5.11
**Mineral Soil Layer (SOC < 17%, n=19)**									
Median	**4.7**	**0.023**	**0.64**	**0.26 (0.29*)**	**1.6**	**0.1**	**19**	**-27.7**	**0.688**
Interquartile Range	*6.3*	*0.042*	*1.2*	*0.2 (0.32*)*	*5.2*	*0.29*	*5.8*	*1.15*	*1.35*
Minimum	1.1	0.0057	0.1	0.081	0.35	0.011	5.8	-30.6	-1.31
Maximum	15	0.21	4.4	1.4	13	0.87	32	-25.1	5.56

**Whole Core (n=178)**									
Median	**33**	**0.41**	**1.4**	**0.088 (0.088*)**	**44**	**2.0**	**20**	**-28.0**	**0.549**
Interquartile Range	*30*	*0.90*	*2.6*	*0.084 (0.085*)*	*10*	*0.70*	*5.3*	*0.81*	*1.35*
Minimum	1.1	0.0057	0.1	0.031	0.35	0.011	5.8	-30.6	-1.35
Maximum	210	28	17	1.4	52	3.3	54	-24.9	5.56

*Median and Interquartile Range without weighing the data. This is included for possible comparison to other studies where R_{HgC} is reported as unweighted data.

Table S3. Results from statistical analysis (one-way ANOVA and Tukey's test for post-hoc analysis) comparing HgT, MeHg, %MeHg and R_{HgTC} between the study sites for the top 50 cm, top 1 m and peat-only core sections (S = Suossjavri, D = Dávva)

	< 50 cm		1 m		Peat-only	
Tested variable	one-way ANOVA	Tukey's post-hoc	one-way ANOVA	Tukey's post-hoc	one-way ANOVA	Tukey's post-hoc
HgT	$p > 0.05$	-	$p > 0.05$	-	$p < 0.05$	$p < 0.05$ (S > D)
MeHg	$p > 0.05$	-	$p > 0.05$	-	$p > 0.05$	-
%MeHg	$p > 0.05$	-	$p > 0.05$	-	$p > 0.05$	-
R_{HgTC}	$p > 0.05$	-	$p > 0.05$	-	$p > 0.05$	-

Table S4. Summary statistics for the three core classes for peat core depth intervals and whole core (median and interquartile ranges are weighted by depth and by core).

		HgT ng/g	MeHg ng/g	MeHg %	R_{HgC} μg Hg/g C	SOC %	N %	C/N	$\delta^{13}C$ ‰	$\delta^{15}N$ ‰
Collapse Fen	**0-50 cm (n=25)**									
	Median	**61**	**1.8**	**6.1**	**0.13**	**46**	**2.1**	**19**	**-28.3**	**1.12**
	Interquartile Range	*39*	*4.7*	*6.5*	*0.11*	*5.9*	*0.89*	*5.2*	*0.641*	*1.45*
	Minimum	20	0.17	0.49	0.048	27	1.5	15	-28.8	-0.498
	Maximum	210	28	17	0.43	50	3.3	30	-27.1	5.11
	**50-100 cm (n=17)**									
	Median	**23**	**0.93**	**3.5**	**0.085**	**42**	**1.7**	**20**	**-28**	**0.705**
	Interquartile Range	*21*	*1.2*	*3.3*	*0.097*	*26*	*0.88*	*4.6*	*0.924*	*0.721*
	Minimum	6.6	0.043	0.42	0.043	2.8	0.15	17	-30.1	-0.502
	Maximum	82	3.3	8.4	0.23	48	2.5	26	-27.9	1.43
	**100-150 cm (n=3)**									
	Median	**37**	**1.3**	**4.5**	**0.087**	**44**	**1.8**	**25**	**-27.8**	**-0.171**
	Interquartile Range	*7.6*	*0.46*	*2.2*	*0.022*	*2.2*	*0.065*	*0.43*	*0.501*	*0.0693*
	Minimum	25	0.69	2.3	0.059	42	1.7	25	-27.8	-0.275
	Maximum	37	1.3	4.5	0.087	44	1.8	25	-27.3	-0.171
	**Whole Core (n=51)**									
	Median	**42**	**1.2**	**3.9**	**0.12**	**44**	**1.8**	**20**	**-28.0**	**0.80**
	Interquartile Range	*51*	*2.7*	*4.3*	*0.12*	*9.8*	*0.66*	*6.2*	*0.68*	*1.18*
	Minimum	2.8	0.023	0.42	0.043	0.35	0.026	13	-30.6	-1.02
	Maximum	210	28	17	1.35	50	3.3	30	-26.0	5.11

Cont. Table S4.

		HgT ng/g	MeHg ng/g	MeHg %	R_{HgC} µg Hg/g C	SOC %	N %	C/N	$\delta^{13}C$ ‰	$\delta^{15}N$ ‰
**Peat Plateau**	**0-50 cm (n=25)**									
	Median	**55**	**0.17**	**0.48**	**0.12**	**47**	**2.3**	**21**	**-27.7**	**0.883**
	Interquartile Range	*43*	*0.55*	*1.7*	*0.093*	*3.3*	*0.85*	*6.5*	*0.821*	*1.82*
	Minimum	23	0.012	0.02	0.046	32	0.87	14	-28.9	-0.562
	Maximum	190	2.4	7.1	0.4	52	3.3	54	-25.4	3.8
	**50-100 cm (n=22)**									
	Median	**29**	**0.18**	**0.55**	**0.082**	**47**	**2.2**	**20**	**-28.3**	**0.162**
	Interquartile Range	*7.4*	*0.34*	*1.4*	*0.057*	*16*	*0.52*	*2.8*	*0.69*	*1.31*
	Minimum	16	0.062	0.19	0.033	28	0.87	17	-30.1	-0.836
	Maximum	55	0.8	3.2	0.18	52	2.7	35	-27.8	1.21
	**100-150 cm (n=10)**									
	Median	**25**	**0.39**	**1.5**	**0.054**	**46**	**1.9**	**23**	**-28.4**	**-0.206**
	Interquartile Range	*5.2*	*0.37*	*1.7*	*0.0096*	*3.8*	*0.75*	*7.2*	*0.477*	*1.63*
	Minimum	19	0.04	0.15	0.043	39	1.5	19	-29	-1.1
	Maximum	29	0.5	2.2	0.075	48	2.5	29	-27.8	0.929
	**Whole Core (n=76)**									
	Median	**28**	**0.20**	**0.70**	**0.074**	**46**	**2.0**	**21**	**-28.0**	**0,395**
	Interquartile Range	*18*	*0.36*	*1.6*	*0.073*	*9.8*	*0.94*	*5.8*	*0.940*	*1,36*
	Minimum	1.1	0.0057	0.020	0.031	0.36	0.11	5.8	-30.1	-1.35
	Maximum	190	2.4	7.1	*0.71*	*52*	*3.3*	54	-24.9	5.56

Cont. Table S4.

		HgT ng/g	MeHg ng/g	MeHg %	R_{HgC} µg Hg/g C	SOC %	N %	C/N	$\delta^{13}C$ ‰	$\delta^{15}N$ ‰
Distant Fen	**0-50 cm (n=21)**									
	Median	**42**	**0.78**	**1.7**	**0.092**	**42**	**2.2**	**18**	**-27.6**	**0.363**
	Interquartile Range	*22*	*1.2*	*2.4*	*0.04*	*6.1*	*0.62*	*2.6*	0.67	1.72
	Minimum	26	0.21	0.63	0.059	38	1.6	16	-28.6	-1.22
	Maximum	64	3.9	11	0.16	48	2.9	26	-26.5	1.55
	**50-100 cm (n=17)**									
	Median	**35**	**0.33**	**0.72**	**0.085**	**37**	**2**	**19**	**-28.1**	**0.245**
	Interquartile Range	*33*	*0.7*	*0.94*	*0.078*	*15*	*0.4*	*3.4*	0.818	1.3
	Minimum	17	0.067	0.15	0.052	27	1.4	17	-28.5	-0.838
	Maximum	57	1.3	3.8	0.15	49	2.9	22	-27.5	1.27
	**100-150 cm (n=8)**									
	Median	**33**	**0.17**	**0.48**	**0.066**	**41**	**1.9**	**21**	**-28.5**	**0.381**
	Interquartile Range	*19*	*0.12*	*0.52*	*0.058*	*8.9*	*0.14*	*3*	0.493	0.892
	Minimum	24	0.057	0.15	0.056	33	1.4	20	-28.8	-1.19
	Maximum	49	0.3	0.93	0.13	49	2.2	29	-28	0.567
	**Whole Core (n=51)**									
	Median	**38**	**0.37**	**0.94**	**0.10**	**41**	**2.1**	**19**	**-28.0**	**0.358**
	Interquartile Range	*20*	*0.74*	*1.3*	*0.056*	*9.6*	*0.78*	*3.3*	0.82	1.35
	Minimum	2.0	0.0081	0.15	0.051	0.49	0.28	14	-28.8	-1.23
	Maximum	64	3.9	11	0.48	49	2.9	29	-26.5	*2.78*

Table S5. Results from statistical analysis (one-way ANOVA and Tukey's test for post-hoc analysis) comparing HgT, MeHg, %MeHg, R_{HgTC} and ancillary parameters between core classes (Cf = Collapse fen, Pp = Peat plateau, and Df = Distant fen) for the top 50 cm, top m and peat-only core sections

	< 50 cm		1 m		Peat-only	
Tested variable	one-way ANOVA	Tukey's post-hoc	one-way ANOVA	Tukey's post-hoc	one-way ANOVA	Tukey's post-hoc
HgT	$p > 0.05$	-	$p > 0.05$	-	$p > 0.05$	-
MeHg	$p < 0.05$	$p < 0.01$ (Cf > Pp)	$p < 0.05$	$p < 0.05$ (Cf > Pp)	$p < 0.05$	$p < 0.05$ (Cf > Pp)
MeHg	$p < 0.05$	$p < 0.05$ (Cf > Pp)	$p < 0.05$	$p < 0.05$ (Cf > Pp)	$p < 0.05$	$p < 0.05$ (Cf > Df)
					$p < 0.05$	$p < 0.05$ (Cf > Pp)
$_{HgTC}$	$p > 0.05$	-	$p > 0.05$	-	$p > 0.05$	-
%SOC	$p > 0.05$	-	$p > 0.05$	-	$p > 0.05$	-
$\delta^{13}C$	$p > 0.05$	-	$p > 0.05$	-	$p > 0.05$	-
%N	$p > 0.05$	-	$p > 0.05$	-	$p > 0.05$	-
$\delta^{15}N$	$p > 0.05$	-	$p > 0.05$	-	$p > 0.05$	-
C/N	$p > 0.05$	-	$p > 0.05$	-	$p > 0.05$	-

Table S6. Results from statistical analysis (two-way ANOVA and Tukey's test for post-hoc analysis) comparing HgT, MeHg, %MeHg, R_{HgTC} and ancillary parameters between core classes (Cf = Collapse fen, Pp = Peat plateau, and Df = Distant fen) and with depth (where "Shallow" refers to the < 50 cm core section and "Deep" refers to the interval > 50 cm to max peat depth)

	Depth interval		Core class		
Tested variable	two-way ANOVA	Tukey's pairwise	two-way ANOVA	Tukey's pairwise	Tukey's pairwise - Interactions
HgT	$p > 0.05$	-	$p > 0.05$	-	-
MeHg	$p > 0.05$	-	$p < 0.05$	$p < 0.05$ (Cf > Df)<br>$p < 0.01$ (Cf > Pp)	$p < 0.05$ (Shallow Cf < Shallow Pp)
%MeHg	$p > 0.05$	-	$p < 0.001$	$p < 0.05$ (Cf > Df)<br>$p < 0.001$ (Cf > Pp)	$p < 0.05$ (Shallow Cf < Shallow Pp)
R_{HgTC}	$p < 0.01$	$p < 0.01$ (Shallow > Deep)	$p > 0.05$	-	-
%SOC	$p < 0.05$	$p < 0.01$ (Shallow > Deep)	$p > 0.05$	-	-
$\delta^{13}C$	$p < 0.01$	$p < 0.01$ (Shallow > Deep)	$p > 0.05$	-	-
%N	$p < 0.05$	$p < 0.01$ (Shallow > Deep)	$p > 0.05$	-	-
$\delta^{15}N$	$p < 0.05$	$p < 0.01$ (Shallow > Deep)	$p > 0.05$	-	-
C/N	$p > 0.05$	-	$p > 0.05$	-	-

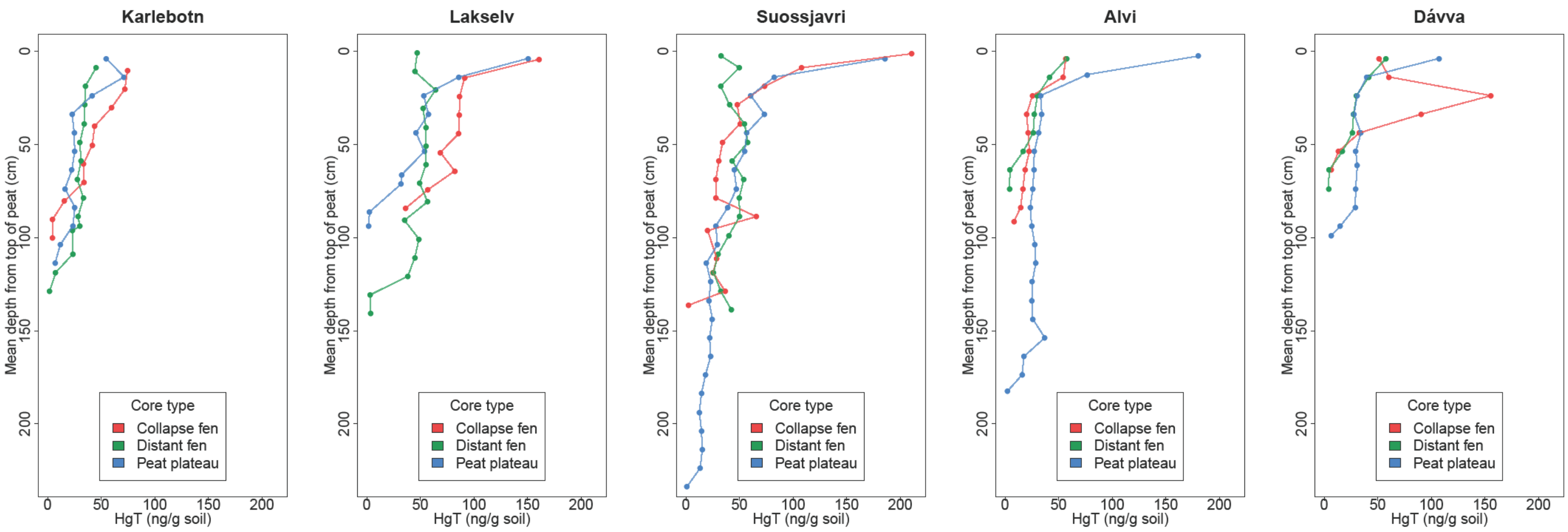

Figure S4. Depth-distribution of total mercury (HgT) concentration in ng g^{-1} soil for each analyzed horizon for each core class and separated by site. Across all classes, HgT concentration tends to decrease with depth.

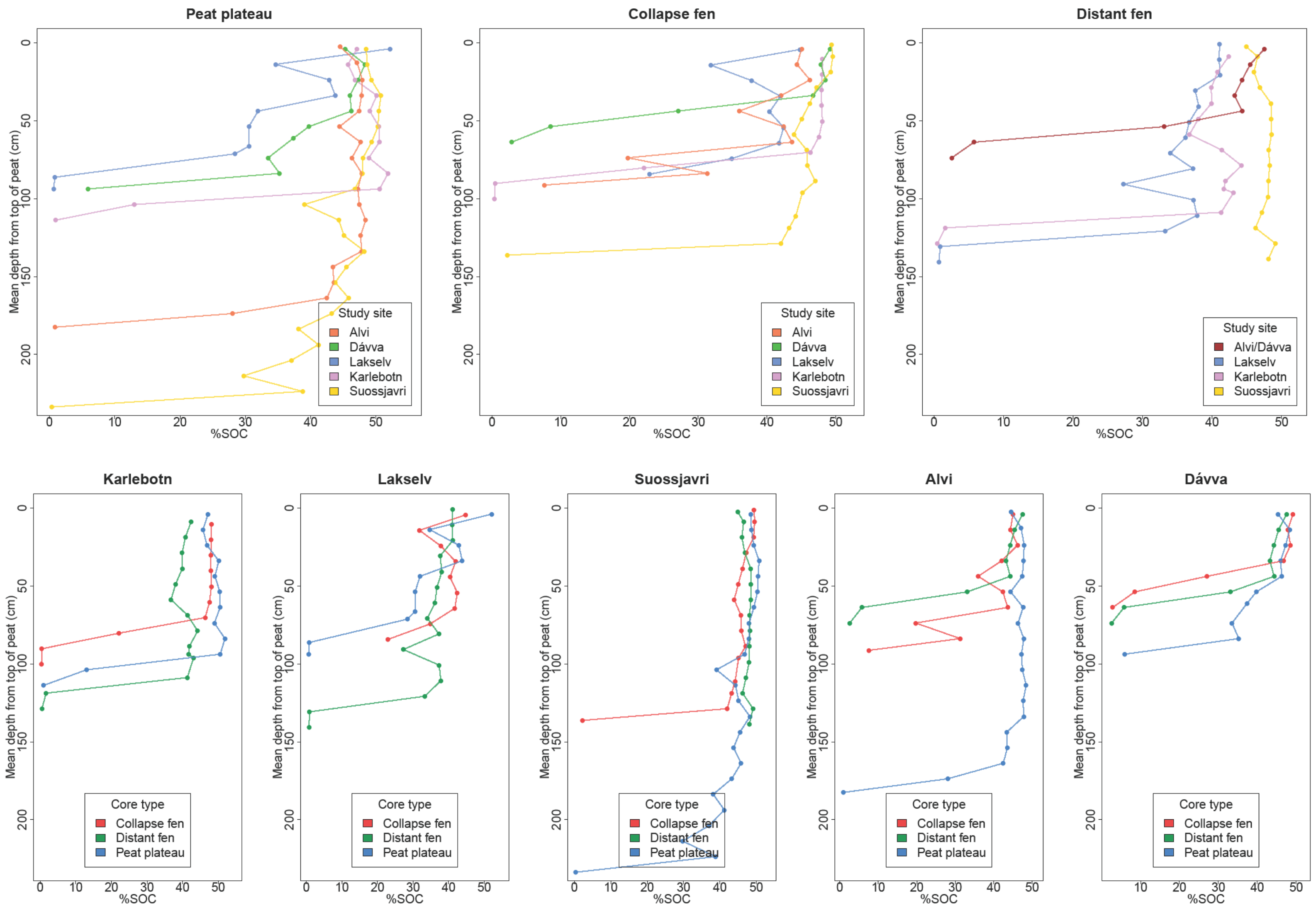

Figure S5. Depth-distribution of the percent soil organic carbon (%SOC) of each analyzed soil horizon separated by core class (top) and by site (bottom). SOC is consistently around 40% for organic soils of the cores and decreases rapidly with increased quantities of minerogenic sediment.

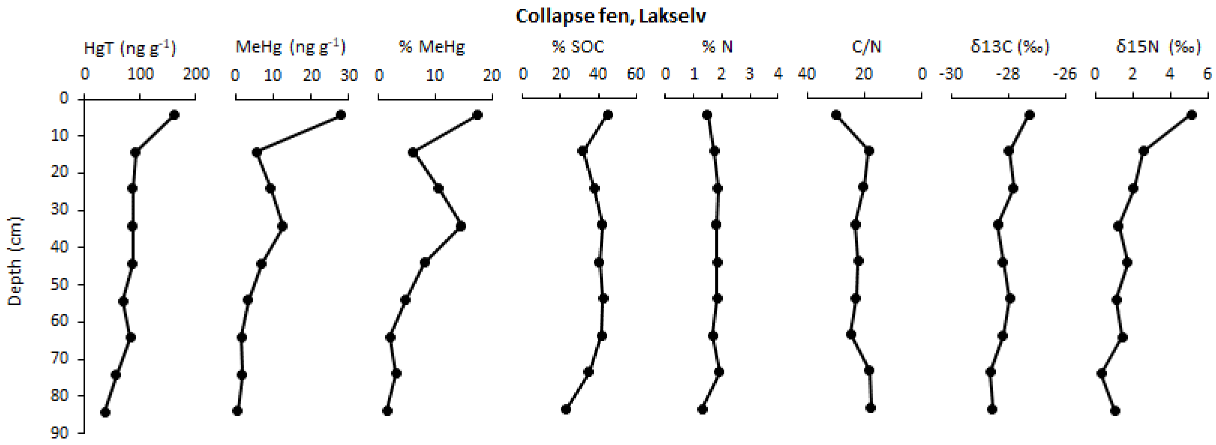
Collapse fen, Lakselv
HgT (ng g-1)
MeHg (ng g-1)
% MeHg
% SOC
% N
C/N
δ13C (‰)
δ15N (‰)
Depth (cm)

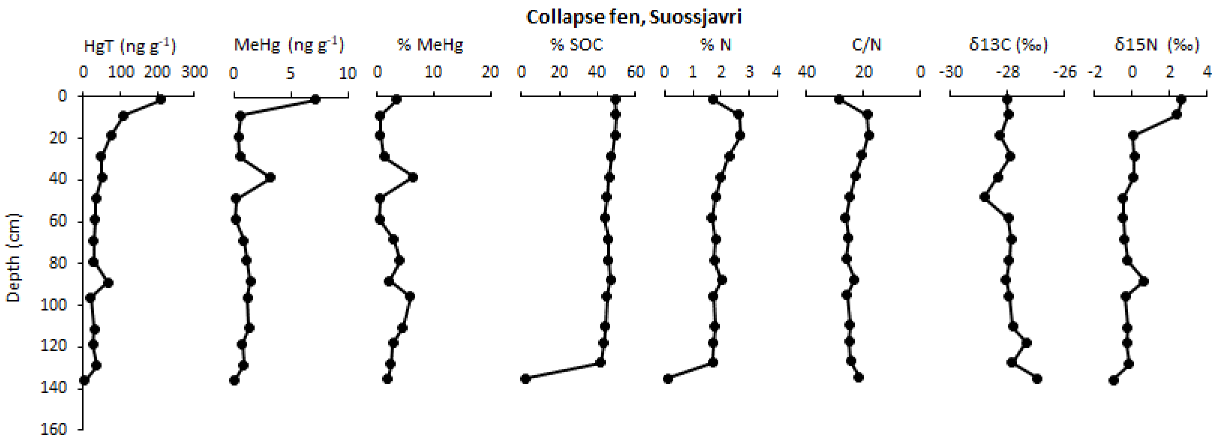
Collapse fen, Suossjavri
HgT (ng g-1)
MeHg (ng g-1)
% MeHg
% SOC
% N
C/N
δ13C (‰)
δ15N (‰)
Depth (cm)

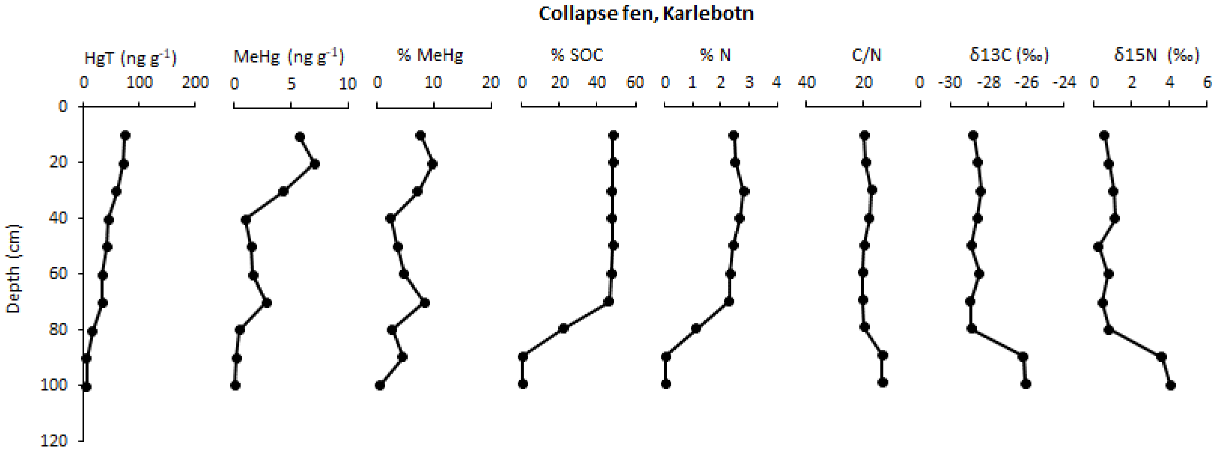
Collapse fen, Karlebotn
HgT (ng g-1)
MeHg (ng g-1)
% MeHg
% SOC
% N
C/N
δ13C (‰)
δ15N (‰)
Depth (cm)

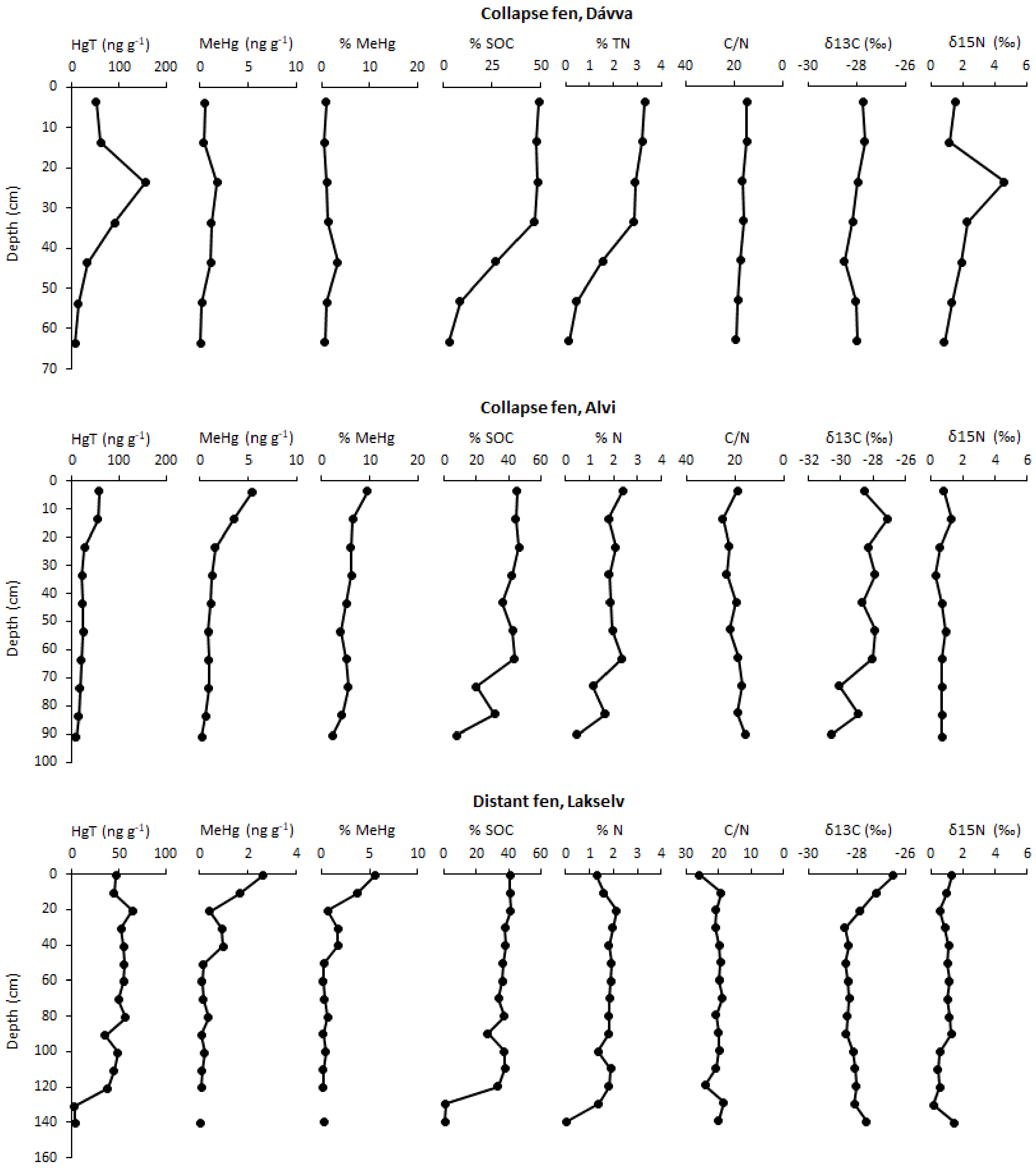

Collapse fen, Dávva
HgT (ng g-1)
MeHg (ng g-1)
% MeHg
% SOC
% TN
C/N
δ13C (‰)
δ15N (‰)
Depth (cm)
Collapse fen, Alvi
HgT (ng g-1)
MeHg (ng g-1)
% MeHg
% SOC
% N
C/N
δ13C (‰)
δ15N (‰)
Depth (cm)
Distant fen, Lakselv
HgT (ng g-1)
MeHg (ng g-1)
% MeHg
% SOC
% N
C/N
δ13C (‰)
δ15N (‰)
Depth (cm)

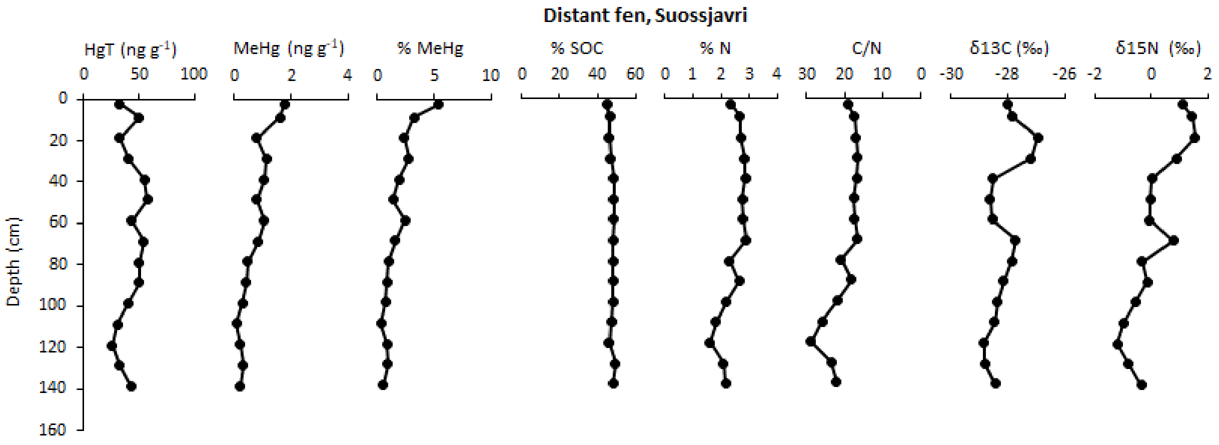
Distant fen, Suossjavri
HgT (ng g-1)
MeHg (ng g-1)
% MeHg
% SOC
% N
C/N
δ13C (‰)
δ15N (‰)
Depth (cm)

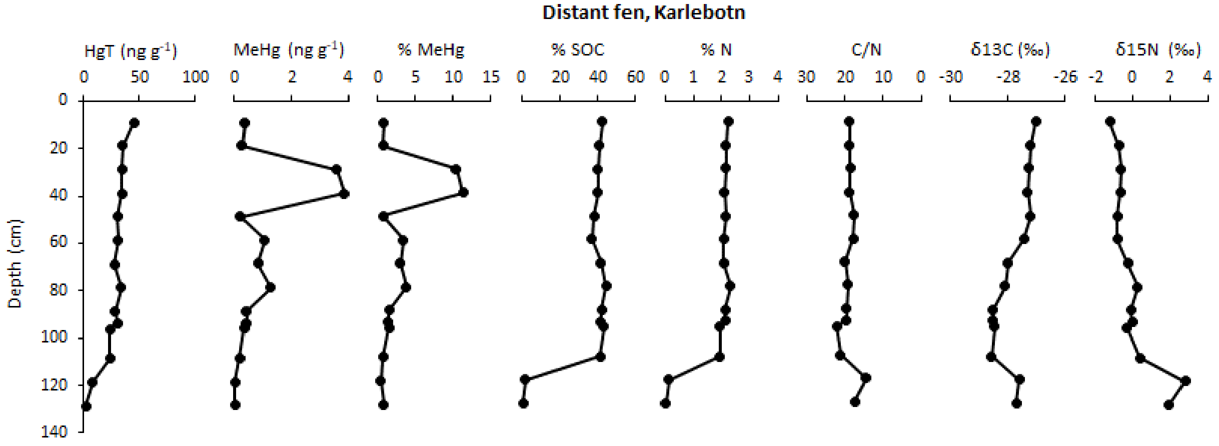
Distant fen, Karlebotn
HgT (ng g-1)
MeHg (ng g-1)
% MeHg
% SOC
% N
C/N
δ13C (‰)
δ15N (‰)
Depth (cm)

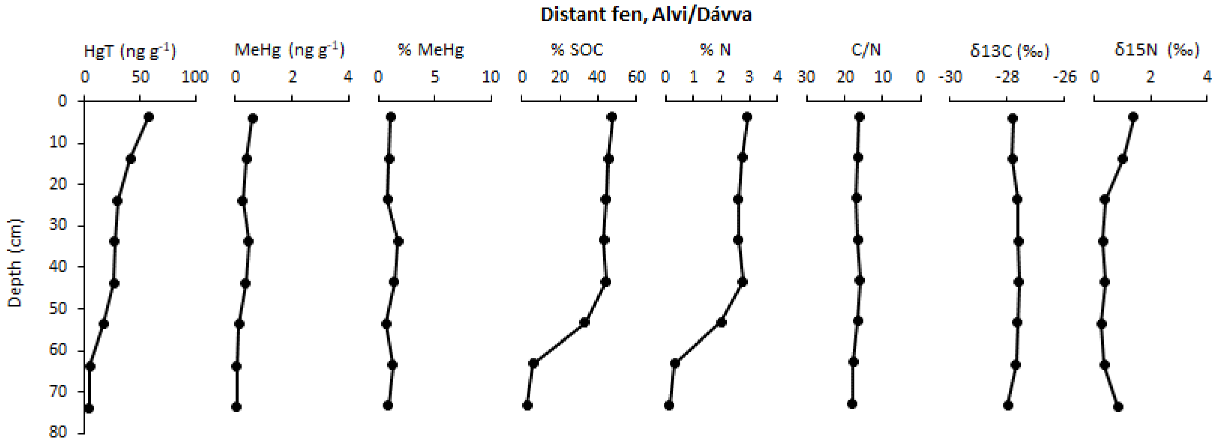
Distant fen, Alvi/Dávva
HgT (ng g-1)
MeHg (ng g-1)
% MeHg
% SOC
% N
C/N
δ13C (‰)
δ15N (‰)
Depth (cm)

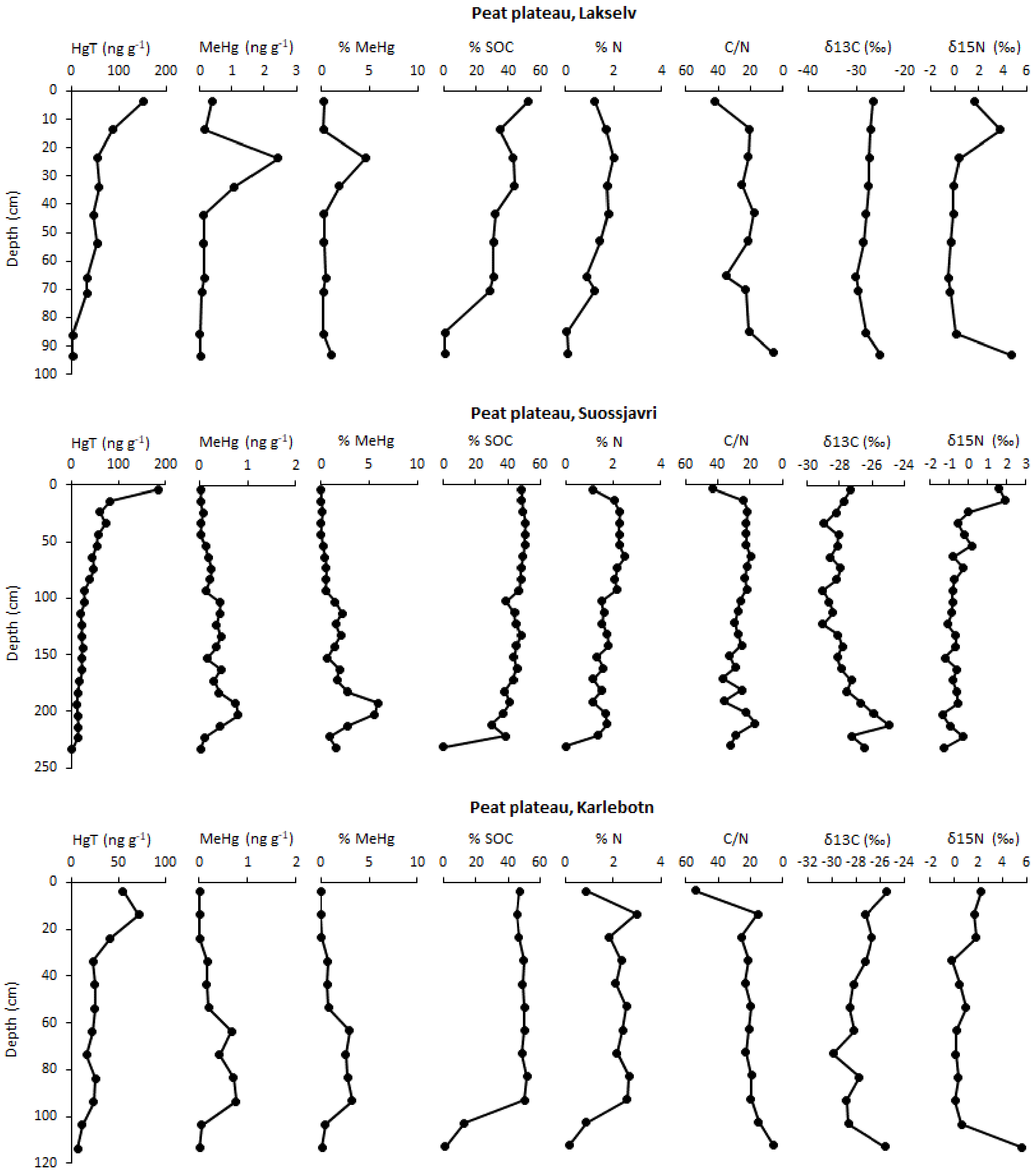

Peat plateau, Lakselv
Peat plateau, Suossjavri
Peat plateau, Karlebotn
HgT (ng g-1)
MeHg (ng g-1)
% MeHg
% SOC
% N
C/N
δ13C (‰)
δ15N (‰)
Depth (cm)

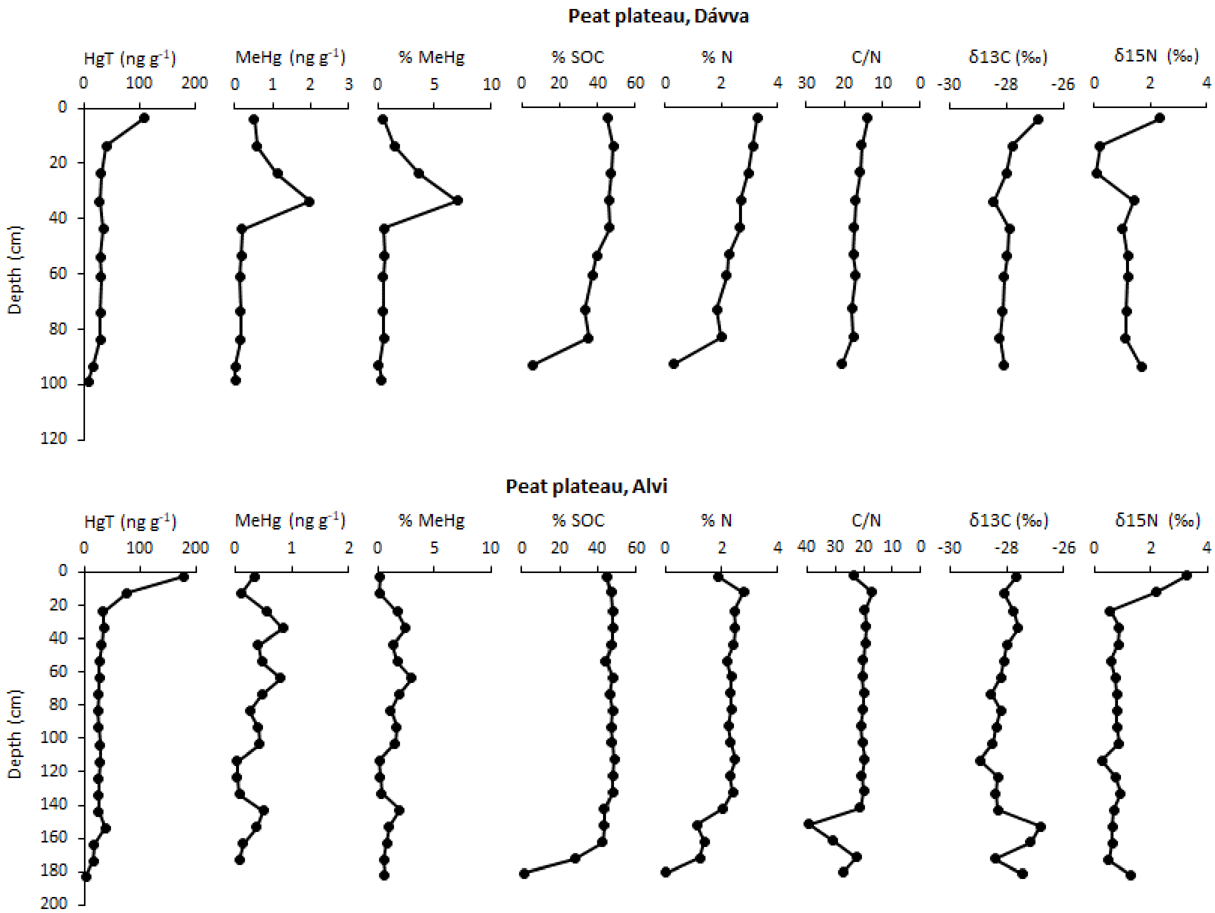

Figure S6. Depth-distribution of HgT, MeHg, %MeHg, SOC, N, C/N, δ13C and δ15N in the 14 cores investigated.

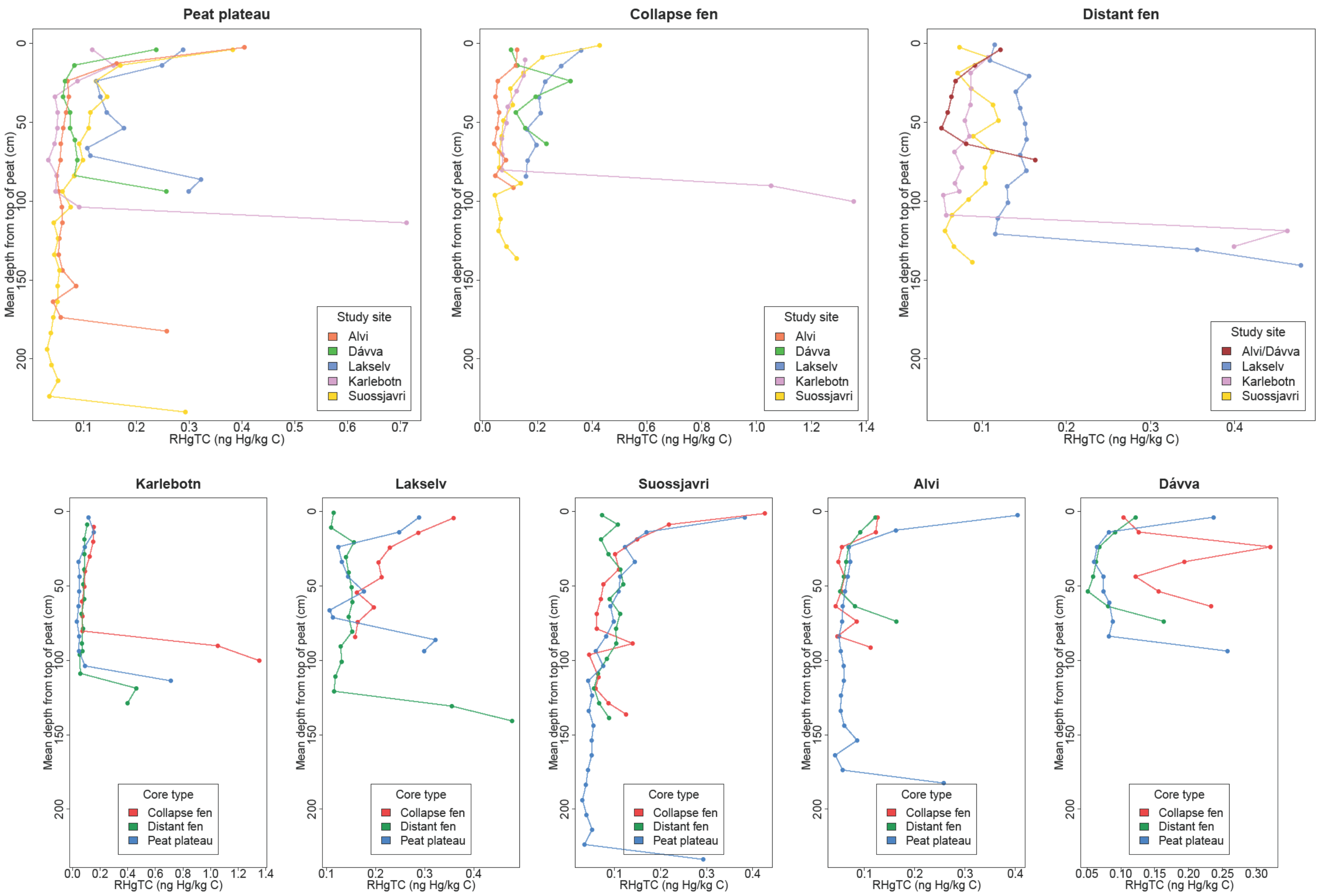

Figure S6. Depth-distribution of total mercury (HgT) concentration normalized to organic carbon (C), i.e. the ratio between HgT and C (R_{HgTC}), for each analyzed soil horizon and separated by core class (top) and site (bottom).

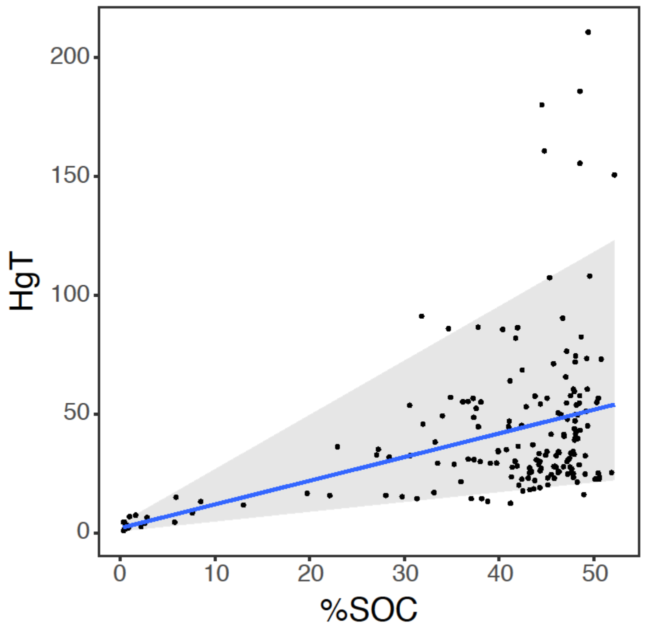

Figure S8. HgT as a function of %SOC (R_{HgTC}), n = 178. The blue line is the median of the data and the gray area is the 90% envelope. Created in R using the ggplot2 package.[12,16]

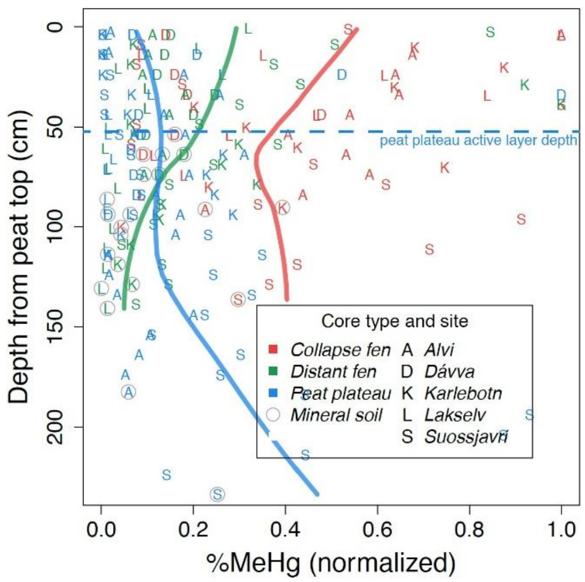

Figure S9. Depth-distribution of %MeHg (normalized to the max value of each site) for each core class, across all sites, with local regression (LOESS).

As noted in the main text, the cores were transported from the field to the lab at temperatures above the ambient soil temperatures (Table S1), but these increased temperatures are unlikely to explain any of the trends observed. We base this assumption on the fact that: i) we observe lower concentrations of MeHg (and %MeHg) in the deeper portion of the active layer where the differences between *in-situ* and transported temperatures were the greatest; ii) we observe differences in %MeHg in the upper layer of the distant and collapse fens, although these were sampled and treated in parallel, and are assumed to have similar *in-situ* temperature (Figures 2 and 3); and iii) we observe no artificial methylation in the control experiment done to test for artificial methylation during transport (described below). Furthermore, the cores were transported intact prior to subdivision in the lab, and if methylation was caused by processes during transport, we would expect the cores to be affected equally across depths (which they are not). For the frozen permafrost layer, "artificial" methylation caused by thaw during transport cannot be categorically ruled out. However, the concentrations of MeHg in the frozen layer were low, suggesting limited methylation during transport.

The test performed to rule out the role of Hg methylation during transportation was done using similar peat samples collected from Abisko, Sweden in 2019 (n=2, the peat material used was kept at -20°C from the sampling day until the experiment). The materials were incubated at 17°C during ~1 week and then analyzed for MeHg. No artificial methylation was detected during the incubation of the two samples. For one of the samples, the concentration of MeHg observed in the incubated sample was ~40% lower in the sample that was not incubated, suggesting some demethylation to occur. This loss is, however, not significant in relation to the 10 and 13 times higher %MeHg we observed in the collapsed fen.

References

(1) Sannel, A. B. K.; Hempel, L.; Kessler, A.; Preskienis, V. Holocene Development and Permafrost History in Sub-Arctic Peatlands in Tavvavuoma, Northern Sweden. *Boreas* **2018**, *47* (2), 454–468. https://doi.org/10.1111/bor.12276.

(2) Brown, J., Ferrians Jr., O. J., Heginbottom, J. A., and Melinkov, E. S. *Circum-Arctic Map of Permafrost and Ground Ice Conditions, Scale 1:10,000,000.*; U.S. Geological Survey, Washington, D. C., 1997.

(3) Gisnås, K.; Etzelmüller, B.; Lussana, C.; Hjort, J.; Sannel, A. B. K.; Isaksen, K.; Westermann, S.; Kuhry, P.; Christiansen, H. H.; Frampton, A.; et al. Permafrost Map for Norway, Sweden and Finland. *Permafr. Periglac. Process.* **2017**, *28* (2), 359–378. https://doi.org/10.1002/ppp.1922.

(4) Sannel, A. B. K.; Kuhry, P. Warming-Induced Destabilization of Peat Plateau/Thermokarst Lake Complexes. *J. Geophys. Res.* **2011**, *116* (G3), G03035. https://doi.org/10.1029/2010JG001635.

(5) Ivanova, N. V., Kuznetsova, I. L., Parmuzin, I. S., Rivkin, F. M. and Sorokovikov, V. A. Geocryological Conditions in Swedish Lapland. In *Proceedings of the 4th Russian Conference on Geocryology*; 2011; pp

77–82.

(6) Borge, A. F.; Westermann, S.; Solheim, I.; Etzelmüller, B. Strong Degradation of Palsas and Peat Plateaus in Northern Norway during the Last 60 Years. *Cryosph.* **2017**, *11* (1), 1–16. https://doi.org/10.5194/tc-11-1-2017.

(7) Kjellman, S. E.; Axelsson, P. E.; Etzelmüller, B.; Westermann, S.; Sannel, A. B. K. Holocene Development of Subarctic Permafrost Peatlands in Finnmark, Northern Norway. *The Holocene* **2018**, *28* (12), 1855–1869. https://doi.org/10.1177/0959683618798126.

(8) Sannel, A. B. K.; Hugelius, G.; Jansson, P.; Kuhry, P. Permafrost Warming in a Subarctic Peatland – Which Meteorological Controls Are Most Important? *Permafr. Periglac. Process.* **2016**, *27* (2), 177–188. https://doi.org/10.1002/ppp.1862.

(9) Martin, L. C. P.; Nitzbon, J.; Aas, K. S.; Etzelmüller, B.; Kristiansen, H.; Westermann, S. Stability Conditions of Peat Plateaus and Palsas in Northern Norway. *J. Geophys. Res. Earth Surf.* **2019**, *124* (3), 705–719. https://doi.org/10.1029/2018JF004945.

(10) NVE: Norges vassdrags- og energidirektorat. Klima.

(11) Heiri, O.; Lotter, A. F.; Lemcke, G. Loss on Ignition as a Method for Estimating Organic and Carbonate Content in Sediments: Reproducibility and Comparability of Results. *J. Paleolimnol.* **2001**, *25* (1), 101–110. https://doi.org/10.1023/A:1008119611481.

(12) R Core Team. A Language and Environment for Statistical Computing. R Foundation for Statistical Computing,. Vienna, Austria 2019.

(13) EPA, U. S. *EPA Method 7473 (SW-846): Mercury in Solids and Solutions by Thermal Decomposition, Amalgamation, and Atomic Absorption Spectrophotometry*; Revision 0. Washington, DC., 1998.

(14) Qvarnström, J.; Frech, W. Mercury Species Transformations during Sample Pre-Treatment of Biological Tissues Studied by HPLC-ICP-MS. *J. Anal. At. Spectrom.* **2002**, *17* (11), 1486–1491. https://doi.org/10.1039/b205246f.

(15) Sannel, A. B. K. Ground Temperature and Snow Depth Variability within a Subarctic Peat Plateau Landscape. *Permafr. Periglac. Process.* **2020**, *31* (2), 255–263. https://doi.org/10.1002/ppp.2045.

(16) H, W. Ggplot2: Elegant Graphics for Data Analysis. Springer-Verlag: New York. 2016.
